# A Series of Novel 1-*H*-isoindole-1,3(2*H*)-dione Derivatives as Acetylcholinesterase and Butyrylcholinesterase Inhibitors: In Silico, Synthesis and In Vitro Studies

**DOI:** 10.3390/molecules29153528

**Published:** 2024-07-26

**Authors:** Edward Krzyżak, Aleksandra Marciniak, Dominika Szkatuła, Klaudia A. Jankowska, Natalia Dobies, Aleksandra Kotynia

**Affiliations:** 1 Department of Basic Chemical Sciences, Faculty of Pharmacy, Wroclaw Medical University, Borowska 211a, 50-556 Wrocław, Poland; aleksandra.marciniak@umw.edu.pl (A.M.); aleksandra.kotynia@umw.edu.pl (A.K.); 2 Department of Medicinal Chemistry, Wroclaw Medical University, Borowska 211, 50-556 Wroclaw, Poland; dominika.szkatula@umw.edu.pl; 3 Student Scientific Club of Medicinal Chemistry, Wroclaw Medical University, Borowska 211, 50-556 Wroclaw, Poland

**Keywords:** isoindoline-1,3-dione, neurodegenerative diseases, acetylcholinesterase, butyrylcholinesterase, synthesis of phthalimide derivatives, molecular docking and dynamics, AChE and BuChE inhibitors, Alzheimer, drug design

## Abstract

The derivatives of isoindoline-1,3-dione are interesting due to their biological activities, such as anti-inflammatory and antibacterial effects. Several series have been designed and evaluated for Alzheimer’s therapy candidates. They showed promising activity. In this work, six new derivatives were first tested in in silico studies for their inhibitory ability against acetylcholinesterase (AChE) and butyrylcholinesterase (BuChE) enzymes. Molecular docking and molecular dynamic simulation were applied. Next, these compounds were synthesized and characterized by ^1^H NMR, ^13^C NMR, FT-IR, and ESI–MS techniques. For all imides, the inhibitory activity against AChE and BuChE was tested using Ellaman’s method. IC_50_ values were determined. The best results were obtained for the derivative I, with a phenyl substituent at position 4 of piperazine, IC_50_ = 1.12 μM (AChE) and for the derivative III, with a diphenylmethyl moiety, with IC_50_ = 21.24 μM (BuChE). The compounds tested in this work provide a solid basis for further structural modifications, leading to the effective design of potential inhibitors of both cholinesterases.

## 1. Introduction

The pathomechanism of Alzheimer’s disease (AD) involves many processes leading to neurodegeneration [1,2]. Accumulation of abnormally folded proteins and peptides induces oxidative stress and the growth of inflammation, which disrupts the proper functioning of neurons [3]. Besides the aggregation of proteins and peptides, changes in neurotransmission also occur in the course of AD. In 1976, Davies and Maloney proposed the cholinergic hypothesis of Alzheimer’s disease [4]. According to this, memory and thinking disorders are caused by the degeneration of cholinergic neurons in the hippocampus and cerebral cortex. The atrophy of cholinergic neurons leads to a decreased acetylcholine level, a very important neurotransmitter, and choline acetyltransferase, the enzyme responsible for its synthesis. Acetylcholine level is regulated by two enzymes: acetylcholinesterase (AChE) and butyrylcholinesterase (BuChE) [5,6]. Both enzymes belong to the class of serine hydrolase enzymes, which form the group of cholinesterases. With regard to AChE, two distinct isoforms can be identified [7]. AChE is responsible for the breakdown of acetylcholine into choline and acetic acid. In the absence of this reaction, the concentration of ACh at cholinergic synapses is increased [8]. Acetylcholinesterase is mainly found on neurons and plays a leading role in the hydrolysis of acetylcholine, while butyrylcholinesterase in the central nervous system is primarily found on glial cells and its role is secondary. The specificity of BuChE for ACh is less than that of AChE. Despite the fact that both BuChE and AChE are 65% homologous in terms of their amino acid sequence, the former is responsible for only 20% of the total cholinesterase activity present in the brain [9]. Studies on Alzheimer’s disease have shown that, in the later stages of the disease, the amount of AChE significantly decreases, and its role is taken over by BuChE [9,10]. AChE and BChE are also present in tissues not related to neurotransmission. Consequently, research is ongoing to identify other functions for these proteins. It has been demonstrated that they are involved in diseases related to lipid metabolism [11].

Donepezil is the most effective, selective, and reversible acetylcholinesterase inhibitor with no hepatotoxicity. Inhibiting the breakdown of acetylcholine restores impaired cholinergic transmission, thereby alleviating moderate and severe symptoms of dementia. Moreover, due to the variety of factors influencing the progression of Alzheimer’s disease, a multi-target strategy seems necessary: the regulation of the transmission of glutamate and amyloid proteins, as well as a neuroprotective effect by inhibiting various inflammatory signaling pathways. Donepezil, as a model compound, became the starting point for subsequent homologues based on its structure, which were tested as potential drugs for Alzheimer’s disease [12,13]. Among them, derivatives of isoindoline-1,3-dione were also studied. A series of 2-(2-(4-Benzylpiperazin-1-yl)ethyl) isoindoline-1,3-dione derivatives were studied by Farani et al. [14]. The initial structure was modified by introducing an additional substituent in the phenyl ring. All compounds showed inhibitory activity against AChE. For the most active derivative, the IC_50_ was 0.91μM. In the next step [15], a carbonyl group was added to the linker between the piperazine group and the phenyl ring. Further modifications were made to the connector [16]. One of the carbon atoms was replaced by a nitrogen atom. For the compound with the highest inhibitory potency, IC_50_ was found as 1.1 μM. A series of 2-(diethylaminoalkyl)-isoindoline-1,3-dione derivatives were investigated as potential AChE and BuChE inhibitors [17]. The alkyl chain was lengthened from three to eight methylene groups. All compounds had significant or moderate AChE inhibitory activity, with IC_50_ values ranging from 0.9 to 19.5 μM, and weak activity against BuChE. The most promising inhibitors were those with six and seven methylene groups (against AChE) and eight methylene groups (against BuChE). Derivatives of isoindoline-1,3-dione with N-benzylpiperidinylamine moiety were synthesized and evaluated as potential inhibitors of AChE and BuChE by Bajda et al. [18]. The length of the alkyl linker and various substituents in the benzyl ring were tested. All compounds were active against AChE (the best IC_50_ was found to be 87 nM), and some of them were also active against BuChE (the best IC_50_ = 7.76 μM). In the works in [19,20], compounds with N-benzylamine and N-methylbenzylamine moiety, connected to phthalimide ring by an alkyl linker or the alkyl linker with benzene ring in chain, were studied. The derivatives showed statistically significant inhibitory activity towards AChE, and some had a balanced activity against both cholinesterases. The IC_50_ for the most active compounds was 34 nM (against AChE) and 0.54 μM (against BuChE). In the study in [21], series of isoindoline-1,3-dione-N-benzyl pyridinium hybrids were synthesized and evaluated for inhibiting activity against AChE. Biological tests indicated that all derivates presented potent inhibitory activity, with IC_50_ values ranging from 2.1 to 7.4 µM. Karim et al. studied three derivatives with a substituted benzyl ring connected directly to the phthalimide fragment without an alkyl linker [22]. In vitro tests showed activity against AChE with IC_50_ values ranging 10–140 μM and activity towards BuChE with IC_50_ values of 11–80 μM.

**Figure 1:**
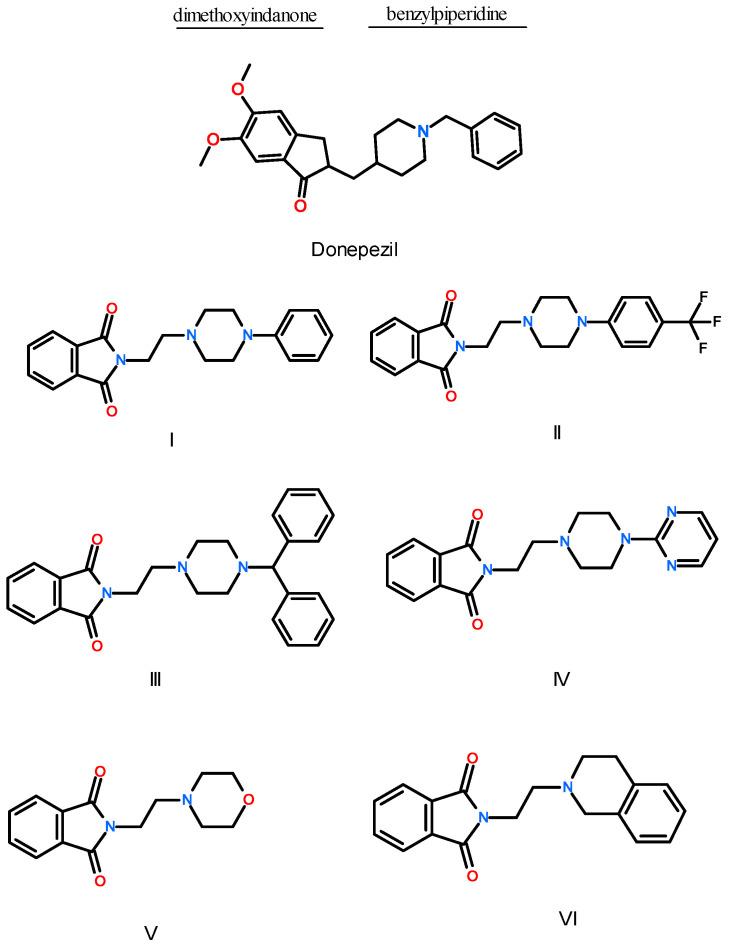
Structure of donepezil and studied compounds: **I**—N-[2-(4-phenyl-1-piperazinyl)ethyl]-1*H*-isoindole-1,3(2*H*)-dione, **II**—N-{2-[4-(4-trifluoromethylphenyl)-1-piperazinyl]ethyl}-1*H*-isoindole-1,3(2*H*)-dione, **III**—N-{2-[4-(4-diphenylomethylo)-1-piperazinyl]ethyl}-1*H*-isoindole-1,3(2*H*)-dione, **IV**—N-{2-[4-(4-pyrimidyn-2-ylo-1-piperazinyl]ethyl}-1*H*-isoindole-1,3(2*H*)-dione, **V**—N-(2-morpholine)ethyl-1*H*-isoindole-1,3(2*H*)-dione, **VI**—N-[2-(3,4-dihydro-1*H*-isoquinolin-yl)ethyl-1*H*-isoindole-1,3(2*H*)-dione.

The subject of our research was the isoindoline-1,3-dione molecule, due to its multidirectional biological effects [23,24]. The acidic properties of the proton at the 2-position imide nitrogen of phthalimide enable easy condensation with multifunctional pharmacophores such as arylpiperazines [25]. As a result of our previous research, we obtained arylpiperazine derivatives with a three-carbon bond and ethylene derivatives linked with an acetyl fragment (two-carbon with a carbonyl group, which lowers the basicity of the nitrogen of the piperazine ring) and imides with the character of Mannich bases. To expand the library of imide compounds with potential biological activity in neurodegenerative diseases, the synthesis of another group of derivatives, which formed the subject of this research, was planned. In this work, six compounds, derivatives of 1-*H*-isoindole-1,3(2*H*)-dione, were designed (Figure 1). The starting structure was the well-known AChE inhibitor, donepezil. The donepezil molecule is built of two moieties, benzylpiperidine group and dimethoxyindanone ring, connected by a short methylene linker. The indadone ring interacts with the enzyme at the top of the active gorge of AChE (PAS region), by π-π contacts with the indole ring of Trp279 [26,27]. In the catalytic anionic site (CAS) of AChE gorge, a nitrogen atom of the piperidine ring forms a cation-π interaction with the phenyl ring of Phe330. Near the bottom of the active site, the benzyl ring interacts via π-π contacts with Trp84 (anionic site). The benzyl ring also interacts with the residues of the oxyanion hole, Gly118, Gly119, and Ala201, forming a hydrogen bond. Three solvent water molecules play an important role in donepezil’s interaction with AChE. In our study, the structure of donepezil was modified as follows: for all compounds, the dimethoxyindanone ring was replaced with a phthalimide ring, and the methylene linker was extended to an ethylene bridge. For compounds **I**–**IV**, the benzylpiperidine moiety was changed to 4-phenylpiperazine, 4-(trifluoromethylphenyl)piperazine, 4-benhydrylpiperazine, and 4-(2-pyrimidyl)piperazine, respectively. For compounds **V** and **VI**, the benzylpiperidine moiety was changed to morpholine and tetrahydroisoquinoline, respectively.

## 2. Results and Discussion

### 2.1. In Silico Interactions Studies

#### 2.1.1. Interactions with Acetylcholinesterase Enzyme (AChE)

Molecular docking and molecular dynamics simulation were applied to study the interactions of AChE (as well as BuChE) with six derivatives of 1-*H*-isoindole-1,3(2*H*)-dione. The crystal structure of AChE with co-crystalized Donepezil, PDB: 1EVE [26], was used for modeling. Molecular docking studies showed that, for all six compounds, the binding affinity (docking score function) for interactions with AChE was negative (Table 1).

**Figure 2:**
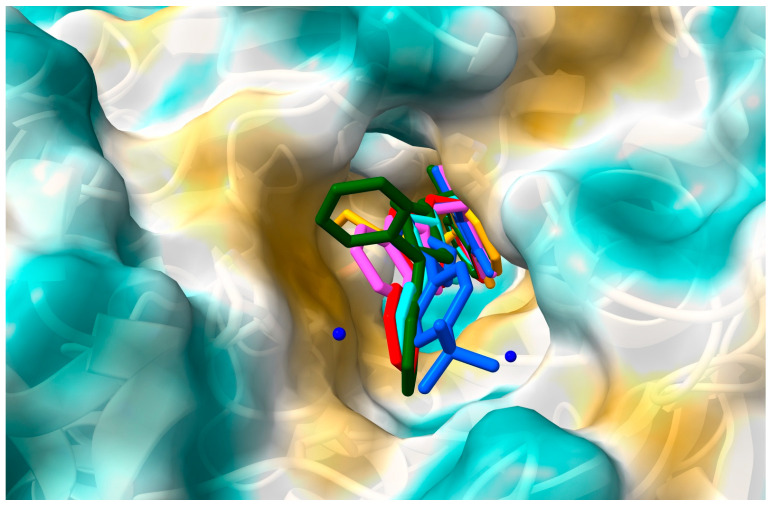
The location of the derivatives **I**–**VI** in the AChE gorge: **I**—red, **II**—blue, **III**—green, **IV**—cyan, **V**—orange, **VI**—magenta.

**Figure 3:**
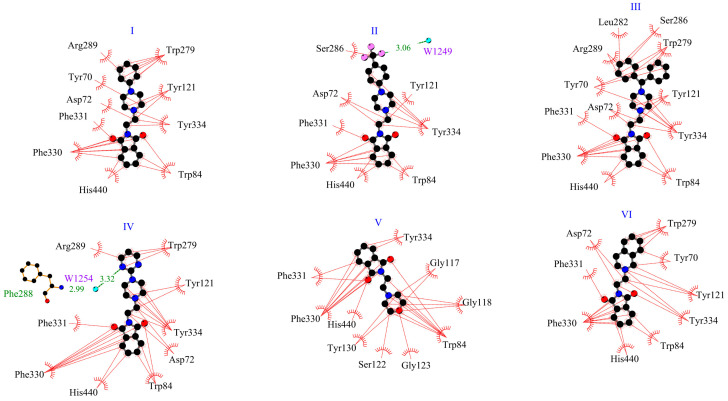
Two-dimensional interaction plot for systems with AChE.

This suggests the formation of stable complexes. Binding affinity values were found between −10.2 kcal/mol for compound **VI** and −8.2 kcal/mol for compound **V**. Using the same docking protocol, for donepezil (redocking from crystal structure 1EVE), the score energy was calculated as −10.9 kcal/mol. Figure 2 shows the position of the six derivatives with the most negative energy in the active AChE pocket. For derivatives **I**–**IV** and **VI**, the phthalimide ring is directed to the inside of the active gorge. The second part of the molecule occupies a position closer to the entrance of the pocket. The derivative **V**, which is considerably shorter than the other compounds, enters deeply into the gorge, and the phthalimide ring is oriented toward the entrance. For the model in which the phthalimide moiety is oriented as for the other derivatives, the binding affinity is less negative, i.e., −7.8 kcal/mol (vs. −8.2 kcal/mol). Figure 3 shows how the tested compounds interacted with AChE. The formation of hydrogen bonds directly with the enzyme was not observed. Only hydrophobic interactions were found. At the entrance to the gorge, molecules **I**–**IV** and **VI** interact with AChE in the peripheral anionic site (PAS), i.e., with Tyr70, Asp72, Tyr121, Trp279, and Tyr334. Interactions occur mainly via π-π contacts with phenylpiperazine, pyrimidylpiperazine, or tetrahydroisoquinoline rings. For all compounds, the enzyme’s catalytic triad (Ser200, Glu327, His4400) interacts through contacts between His330 and the isoindole ring. For all compounds, interactions were also observed at the anionic site (Trp84, Tyr130, Phe330, Phe331). The phthalimide group and Trp 84 (π-π), Phe330 (π-π), and Phe331 (π-π) are involved in hydrophobic contacts. Only for derivative **V** were interactions with residues from the oxyanion hole (Gly118, Gly119, Ala201) observed, i.e., between the morpholine ring and Gly118. For none of the compounds were direct interactions with residues from the acyl pocket (Phe288, Phe290) observed. However, for compound **IV**, a hydrogen bond is formed with Phe288, through a water molecule W1254.

**Figure 4:**
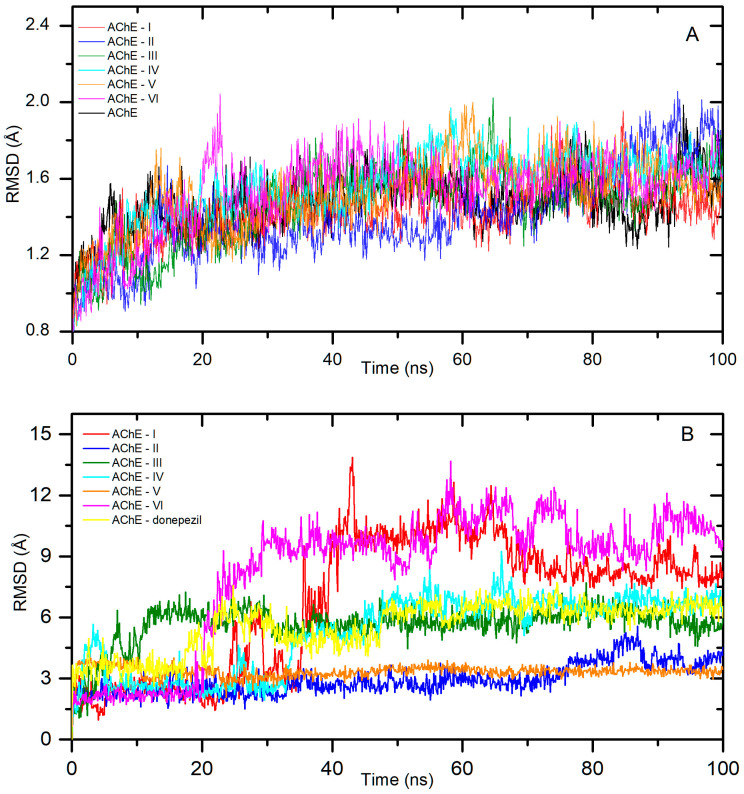
Backbone RMSD (**A**) and the ligand RMSD (**B**) plots after least square fitting to the protein backbone for systems with AChE.

The best pose from the molecular docking studies, with the most negative binding affinity, was taken as a starting point for molecular dynamic studies. The simulation was run for a period of 100 ns to confirm the stability of the complexes and calculate the free binding energy. Figure 4A shows the backbone RMSD plot after the least square fitting to the protein backbone for all systems. All complexes stabilized quite quickly, i.e., after about 10 ns for AChE-I, II, 15 ns for AChE-IV, V, 25 ns for AChE-VI, and 30 ns for AChE-III. The fluctuations were low with an average RMSD of 1.4 ± 0.1 Å, 1.4 ± 0.2 Å, 1.6 ± 0.1 Å, 1.6 ± 0.1 Å, 1.5 ± 0.1 Å, and 1.6 ± 0.1 Å for **I**–**VI**, respectively. Figure 4B shows a ligand RMSD plot after the least square fitting to the protein backbone for all systems. It displays the structural drift of the ligand during the MD simulation. Complexes with compounds **II** and **V** were very stable throughout the simulation period. The averaged root mean square deviation (RMSD) was calculated as 2.9 ± 0.7 Å for **II**, and 3.3 ± 0.3 Å for **V**. Slightly higher fluctuations were observed for systems with ligands **III** and **IV**. The AChE-III system reached stabilization after about 30 ns, and the average RMSD value up to 100 ns was 5.9 ± 0.5 Å. The averaged ligand RMSD value for AChE-IV after stabilization was calculated as 6.7 ± 0.5 Å. The greatest drift from the initial position was shown for derivatives **I** and **VI**. After about 40 ns for **I** and 30 for **VI** to the end of the simulation, the average RMSD value was 9.2 ± 1.2 Å and 10.2 ± 1.0 Å, respectively. In the next step, the binding free energy was calculated from MD complex trajectories, using the gmx_MMPBSA tool [28]. The results are given in Table 1. The ΔG_bind_ was found to be −12.87 ± 1.66, −15.15 ± 1.29, −15.71 ± 2.37, −7.08 ± 1.43, −7.35 ± 1.74, and −14.34 ± 2.02 kcal/mol for systems **I**–**VI**, respectively. For all systems, the values were negative, indicating spontaneous complex formation.

#### 2.1.2. Interactions with Butyrylcholinesterase Enzyme (BuChE)

**Figure 5:**
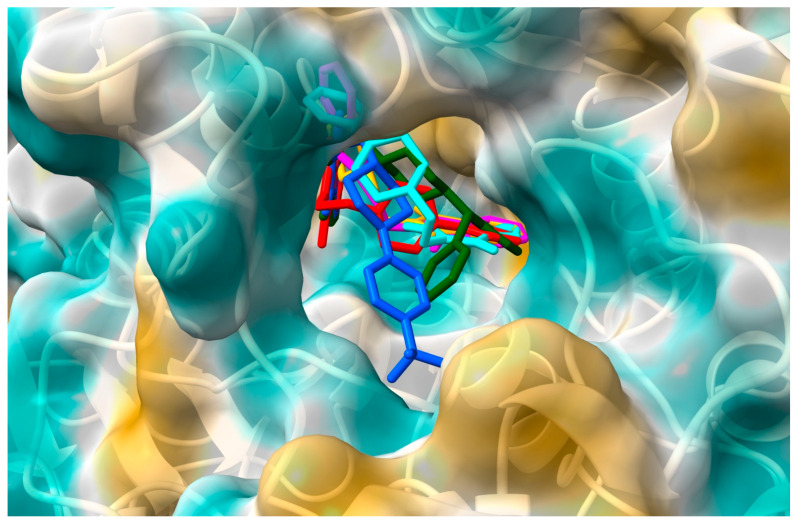
The location of the derivatives **I**–**VI** in the BuChE gorge: **I**—red, **II**—blue, **III**—green, **IV**—cyan, **V**—orange, **VI**—magenta.

**Figure 6:**
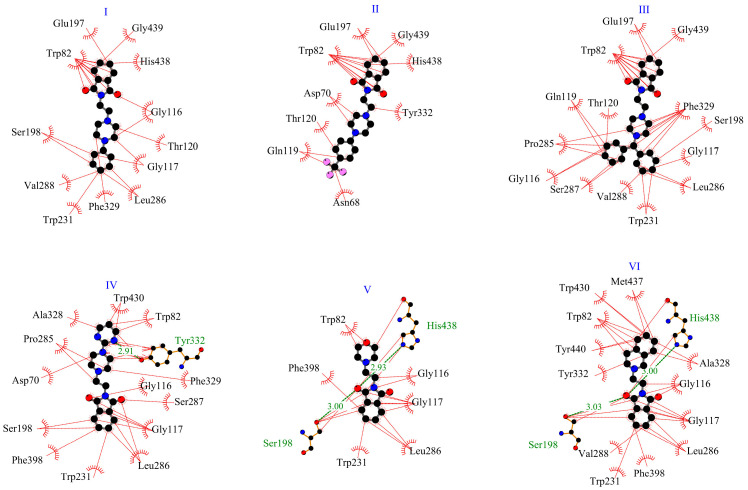
Two-dimensional interaction plot for systems with BuChE.

The crystal structure of BuChe 1P0I [29] was chosen for modeling. Similarly to the previously described interactions with AChE, all tested complexes showed negative binding affinity (Table 1). The best result, −11.0 kcal/mol, was obtained for the derivative **III**, with benhydrylpiperazine moiety. For the other derivatives, the scoring function ranged from −8.3 to −10.2 kcal/mol. Figure 5 presents the position of the studied compounds in the active pocket of BuChE. The enzymatic pocket of butyrylcholinesterase consists of the same parts as AChE, i.e., CAS and PAS. However, it is much wider, so it is also available for larger molecules. And for smaller molecules, the rotation possibilities are greater. For derivatives **I**–**III**, for the most negative binding energy, the isoindole-1,3-dione fragment enters deeply into the gorge. For derivatives **IV-VI**, the molecules are oriented in the opposite direction. Figure 6 presents 2D interaction plots. For the BuChE-IV system, a hydrogen bond is formed between Tyr332 (PAS site) and the pyrimidylpiperazine fragment. For the BuChE-V and BuChE-VI complexes, two hydrogen bonds are formed. The residues His438, Ser198 (acyl pocket), and the oxygen from the phthalimide fragment are involved. In the enzyme gorge, hydrophobic interactions with the catalytic triad, anionic site, acyl pocket, oxyanion hole, and peripheral anionic site were observed. At the entrance (PAS site), derivative **VI** interacts with BuChE via contact (π-alkyl) between Tyr332 and the tetrahydroisoquinoline ring. Compounds **II** and **IV** interact with Asp70 by π-alkyl contacts with the piperazine fragment and with Tyr332 via π-alkyl contact with the linker or pyrimidylpiperazine group. For all derivatives, interactions (π-alkyl, or π-π) with a catalytic triad (Ser198, His438) and anionic site (Trp82, Ala328, Phe329) were observed. In addition, for all systems except BuChE-II, various kinds of hydrophobic contacts in acyl pocket (Leu286, Val288) and oxyanion hole (Gly116, Gly117) were found.

**Figure 7:**
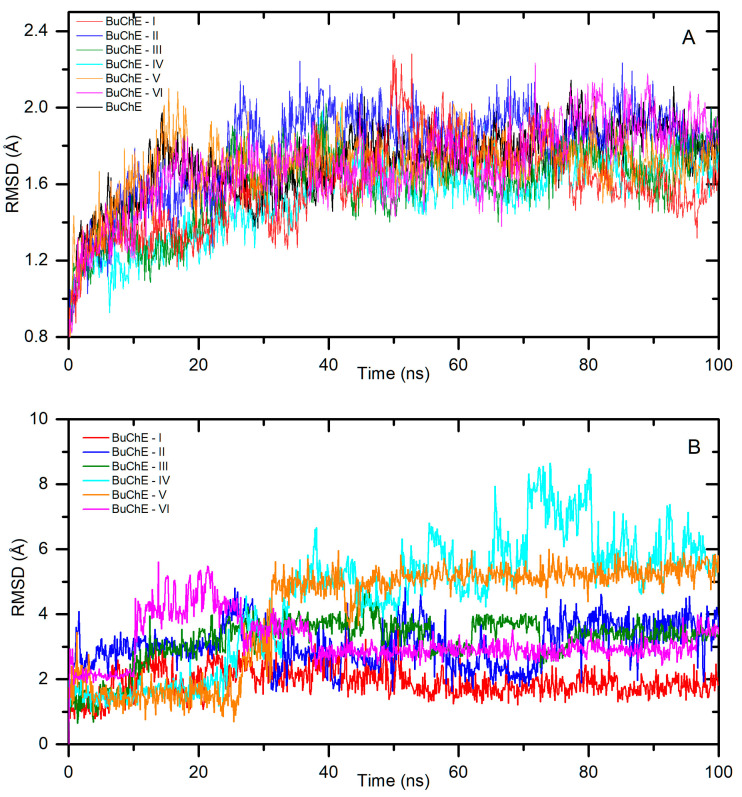
Backbone RMSD (**A**) and the ligand RMSD (**B**) plots after least square fitting to the protein backbone for systems with BuChE.

Next, a molecular dynamic simulation was performed. Figure 7A presents the backbone RMSD plot after the least square fitting to the protein backbone. The systems reached stabilization after about 20 ns (BuChE-VI), 30 ns (BuChE-II, III), 40 ns (BuChE-IV, V), and 50 ns (BuChE-I). The fluctuations were low, with average RMSDs of 1.7 ± 0.1 Å, 1.9 ± 0.1 Å, 1.7 ± 0.1 Å, 1.6 ± 0.1 Å, 1.7 ± 0.1 Å, and 1.8 ± 0.2 Å for **I**–**VI**, respectively. Figure 7B shows a ligand RMSD plot after the least square fitting to the protein backbone for all systems. The smallest drift from the starting position was observed for the derivative **I,** with an average value of RMSD = 2.0 ± 0.4 Å. Compound **VI** in the initial period of the simulation showed insignificant fluctuations, with an RMSD of 3.5 to 5.0 Å. After about 40 ns, it reached stability, and the average value of the structural drift was 2.9 ± 0.2 Å. In the BuChE-II and BuChE-III complexes, ligands were stable after about 30 ns. The average RMSD value to the end of the simulation period was 3.1 ± 0.7 Å and 3.5 ± 0.4 Å, respectively. For the systems with smaller derivatives, i.e., **IV** and **V**, the RMDS was slightly higher, but still at a low level. The complexes were stable after about 40 ns, with the average ligand RMSD value to the end of the simulation period of 5.8 ± 1.0 Å and 5.2 ± 0.4 Å, respectively. The calculated binding free energies are given in Table 1. The ΔG_bind_ was calculated as −13.78 ± 1.42, −13.94 ± 1.71, −15.41 ± 1.81, −7.18 ± 1.37, −1.91 ± 0.51, and −7.03 ± 0.94 kcal/mol for systems **I**–**VI**, respectively. For all systems, the values were negative, indicating spontaneous complex formation. However, system BuChE-V was slightly negative. Derivative **V** was the smallest of those tested and the active BuChE-V pocket was wide, which may have caused a problem with the formation of a stable complex.

Molecular docking and molecular dynamics simulations showed the inhibitory potential of the tested derivatives against both AChE and BuCHE. To confirm this, we decided to synthesize all the compounds and then perform in vitro tests.

### 2.2. Synthesis of Compounds ***I***–***VI***

**Scheme 1:**
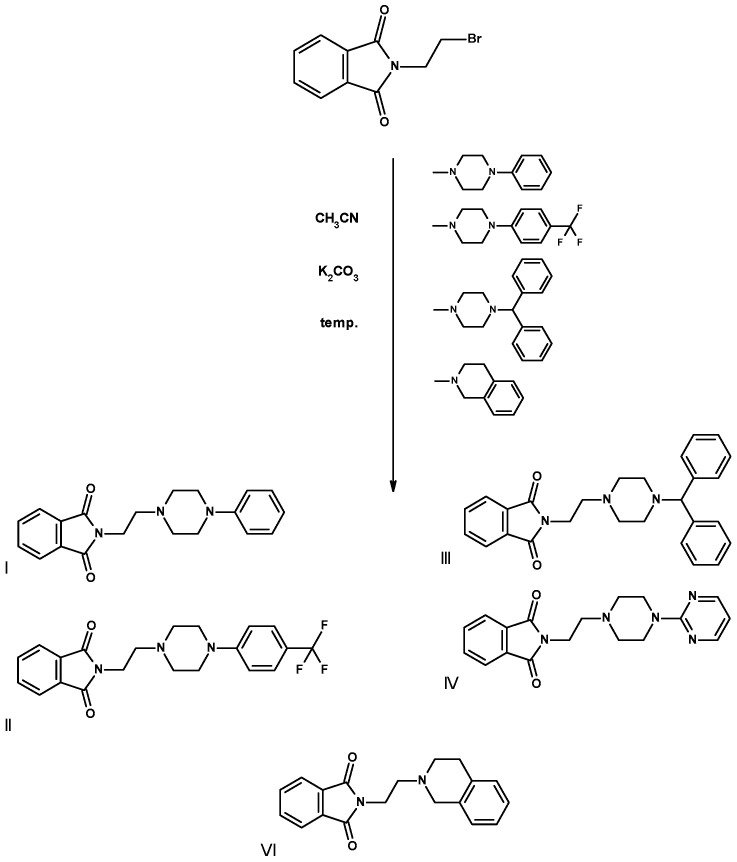
Method of synthesis of final compounds **I**–**IV**, condensation of 2-(2-bromoethyl)phthalimide with cyclic amines using anhydrous potassium carbonate for 5 h during circumference at the boiling point of acetonitrile.

**Scheme 2:**
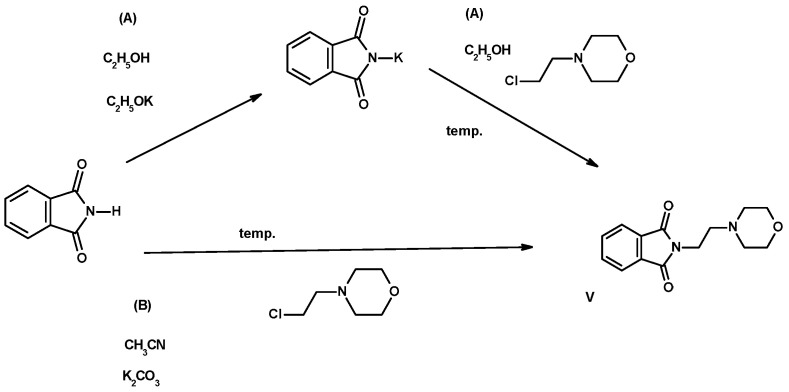
Method for the synthesis of imide V, by condensation of the potassium salt of phthalimide with N-(2-chloroethyl)morpholine at the boiling point of ethanol (method A) using anhydrous potassium carbonate in boiling acetonitrile (method B).

The final compounds **I**–**IV**, **VI** (Scheme 1), and **V** (Scheme 2) were obtained with very good yields as a result of one-step condensation from products available on the market from pharmaceutical companies (Table 2). 

The reactions were carried out in solutions of anhydrous, high-purity solvents (acetonitrile for HPLC and anhydrous 99.96% ethanol) at boiling temperature for 5 h in the presence of anhydrous potassium carbonate, previously micronized by grinding in a porcelain mortar. In the case of imides **I**–**IV**, **VI**, the starting substrate was N-(2-bromoethyl)phthalimide, condensed with the appropriate aryl(heteroaryl)piperazines (**I**–**IV**) and 1,2,3,4-tetrahydroisoquinoline (**VI**), which is a bioisosteric analogue of arylpiperazine [30,31]. Imide **V** was obtained by reacting phthalimide with N-(2-chloroethyl)morpholine, while heating the substrates in anhydrous ethanol in the presence of potassium ethoxylate for 10 h (method A, Scheme 2). This was a reaction with a low yield of 47.20%. In the second method (method B, Scheme 2), a higher yield of the final product **V** was obtained. After releasing N-(2-chloroethyl)morpholine from hydrochloride in the reaction with potassium ethoxylate, the ethanol solution was filtered from the inorganic substance (KCl) and the solvent was completely evaporated under vacuum. The resulting oil was dissolved in acetonitrile, similarly to the case of imides **I**–**IV**, **VI**, condensed on the surface of micronized potassium carbonate with isoinoline-1,3-dione. The efficiency of the synthesis carried out in acetonitrile in the presence of K_2_CO_3_ was significantly higher than in ethanol (86.54%), similarly to the remaining derivatives **I**–**IV**, **VI** (49.79–86.54%). Detailed data are presented in Table 2. The use of micronized potassium carbonate as a K^+^ ion donor increased the effectiveness and shortened the process time. The final compounds were crystallized from ethanol with the addition of a small amount (approximately 5% of the substrate weight) of activated carbon granules. The use of small portions of solvent and preliminary cleaning with granulated activated carbon limited the amount of post-reaction residues in the form of used eluents. In this way, the purification procedure by column chromatography and the production of environmentally harmful organic solvents were not necessary.

Classic analytical methods were used to confirm the structure of compounds **I**–**VI**. Elemental analyses were performed for two compounds, and the error of the theoretical and found values for C, H, N was 0.03%, 0.04%, and 0.06% for imide **I** and 0.14%, 0.32%, and 0.19%, respectively, for imide **II**. In the case of compounds **IV**–**VI**, analyses were not performed, due to the correct indications of the mass spectrometry analysis performed for all compounds. Particularly noteworthy is the high purity of the final products **IV**–**VI**, where the difference between the theoretical values was insignificant.

Using the Bruker’s DataAnalytics program, a computer simulation of the expected compound and its adducts was performed and the value error between the found *m*/*z* and the calculated *m*/*z* was determined. The following error values were recorded: for the molecular ion [L+H]^+^, it was 17.25 ppm (**I**), 13.86 ppm (**II**). In the case of imides **IV**–**VI**, the error value between the theoretical mass and the mass read from the mass spectrum was 2.37 ppm (**IV**), 1.15 ppm (**V**), and 1.77 ppm (**IV**), which indicates the significant purity of the final product obtained in the recrystallization process using activated carbon in ethanol. Additional purification was deemed unnecessary. This was confirmed by the NMR results (^13^C and ^1^H). Signals from the ethylene linker visible in the ^13^C spectrum (in high field, preceding the CDCl_3_ signal) and signals characteristic of the carbon of the benzene ring in the range from 123 ppm to 157.6 ppm confirmed the structure of the final products. Signals corresponding to the carbon atoms of the C=O groups of the imide ring (in the range from 161 ppm to 168.38 ppm for subsequent products—^13^C NMR spectrum) and characteristic infrared peaks (FT-IR) in the range 1700–1780 cm^−1^ (Table 2) confirmed that the N-alkyl derivatives were obtained in accordance with the original assumptions.

### 2.3. In Vitro Studies

To determine the inhibitory properties of the tested compounds against acetylcholinesterase and butyrylcholinesterase, Ellman’s method was used [32]. Evaluations were carried out for six concentrations of tested compounds (10^−4^–10^−9^ mol/dm^3^). Donepezil was used as the reference inhibitor. For each system, measurements were carried out three times. Dose–response curve functions were fitted to the obtained results. Next, the IC_50_ (concentration of the test compound inhibiting 50% of cholinesterase activity) was calculated. The results are presented in Table 3.

In systems with AChE, for the reference inhibitor donepezil, the obtained IC_50_ = 0.04 ± 0.01 µM correlated very well with literature values [15,16]. The highest inhibitory activity was shown by derivative I (with a phenyl substituent at position 4 of piperazine) and derivative **VI** (with a tetrahydroisoquinoline moiety), 1.12 ± 0.30 µM and 1.14 ± 0.17 µM, respectively. The introduction of the -CF_3_ substituent in the para position (**II**) and the change of the phenyl ring to a diphenylmethyl substituent (**III**) slightly reduced the activity of the compound. IC_50_ were calculated as 2.31 ± 0.36 μM and 1.94 ± 0.43 μM, respectively. Derivative **IV**, with a pyrimidyl ring, showed about four-times less activity compared to the derivative with a benzene ring (I). The lowest activity, with IC_50_ = 16.20 ± 2.37 µM, was obtained for the derivative with a morpholine ring (**V**). The inhibiting potential of the tested compounds against AChE was at a similar level to other phthalimide derivatives. In the works of Farani et al. [14,15], for the most active molecule of the series of synthesized compounds, the IC_50_ was 1.1μM and 0.91 μM, respectively. In the work in [21], the activity of the derivatives was in the range of 2.1 to 7.4 μM, and in the work in [22] between 10 and 140 μM.

Inhibitory activity against BuChE of imides **I**–**VI** was determined in the same way as for AChE. The calculated IC_50_ values are given in Table 3. The IC_50_ for donepezil was found to be 3.29 ± 0.85 µM. This value is in good agreement with the values reported in other works [17,22]. For all compounds, the activity against BuChe was lower than against AChE. The best activity was found for derivative **III**, a compound with a diphenylmethyl moiety with IC_50_ = 21.24 ± 4.84 µM. Slightly lower activity was shown by compounds with a phenyl group at position 4 of piperazine (**I**, **II**), 24.67 ± 1.74 µM and 21.71 ± 3.78 µM, respectively. The additional group -CF_3_ in the para position did not affect the activity of the compound. Replacing the benzene at the 4-position of piperazine with a pyrimidyl ring or a tetrahydroisoquinoline moiety reduced the activity of the imides (Table 3). The derivative **V**, with a hydrogenated morpholine ring, showed low activity. The determined IC_50_ was greater than 1000 µM. The low inhibitory activity against BuChE was probably due to the size of the molecule. The slightly larger active pocket than for AChE does not allow it to bind effectively to the CAS and PAS sites. IC_50_ values at the micromole level, similar to those for the tested compounds, also showed other isoindoline-1,3-dione derivatives. In the work in [18], the best derivative showed activity with 7.76 μM. For the series of compounds tested by Karim et al., the IC_50_ was 11–80 μM [22].

In general, compounds that fit well into both the CAS and PAS areas have high inhibitory activity against AChE and BuChE. The presence of interactions with aromatic parts of amino acids is very important is, i.e., π-π stacking and cation-π interactions. Activity also increases when hydrogen bonds are formed at the same time. Such interactions occur in well-known and described inhibitors, such as Donepezil [26], Tactrin [33] or Rivastigmine [34]. The activity of the compounds tested in this work also resulted from this type of contact. For derivatives with similar activity against AChE, i.e., **I**–**IV** and **VI**, π-π stacking interactions were found, both in the CAS (with Trp84, Phe330) and PAS (with Trp279, Tyr334) areas. For derivative **V**, with lower activity, π-π stacking interactions were only observed in the CAS site. The formation of hydrogen bonds could perhaps increase the activity of the tested derivatives. In our next work, we plan to optimize the structure by introducing substituents in the aromatic ring capable of forming hydrogen bonds. For interactions with BuCHE, a higher activity was found for derivatives **I**–**III**. These compounds interact with the enzyme, among others, via π-π stacking contacts with Trp82 at the CAS site. These π-π stacking interactions were not observed for imides **IV** and **VI**, whose activity is lower. In BuChE, six of the fourteen aromatic amino acids included in the active site are replaced by aliphatic or polar amino acids. The PAS site in the BuChE enzyme does not have a fragment composed of aromatic groups, Tyr-Tyr-Trp. Therefore, the presence of hydrogen bonds in the CAS and PAS sites is advantageous. No hydrogen bond formation was observed for derivatives **I**–**III**. Their presence would perhaps increase the activity. For derivatives **IV** and **VI**, hydrogen bonds were observed; however, it seems that the main reason for the lower activity of derivatives **IV** and **VI** is the lack of π-π stacking interactions.

### 2.4. Blood–Brain Barrier and Toxicity

Due to the lack of a possibility to determine experimental penetration of the BBB and toxicity in these studies, we decided to include the predicted values in this work. The probability that the tested imides would cross the blood–brain barrier is high, approximately 0.98 (Table 4). Such results suggest that the tested compounds can cross blood–brain barriers. This is particularly important for potential drugs whose therapeutic target is the central nervous system (CNS). For the molecular structure, the important parameters are the topological polar surface area (TPSA), the logarithm of the n-octanol/water distribution (logD, logP), and molecular mass (MW). The rules for CNS drugs are TPSA < 70 Å^2^, logD between 1 and 3, MW < 450g/mol. For the imides tested, these rules were met. All derivatives should show no carcinogenicity, immunotoxicity, mutagenicity, or cytotoxicity. The predicted LD_50_ classified the tested compounds as toxicity class 4 or 5. Class IV means non-toxic substances, harmful if swallowed, and class V means non-toxic substances, may be harmful if swallowed. This lack of toxicity shows the high safety of using the tested compounds as potential drugs.

## 3. Materials and Methods

### 3.1. Chemistry

#### 3.1.1. Instrumentation and Chemicals

Commercially available chemical reagents with high purity (95–99%) were used for the synthesis. N-(2-bromoethyl)phthalimide, (>98%, C_10_H_8_BrNO_2_, molecular weight (MW) = 254.09, melting point (MP) = 80–84 °C, Alfa Aesar, Ward Hill, MA, USA; 1-phenyl-4-piperazine (>98%, C_10_H_14_N_2_, MW = 162.24, MP = 17–19 °C; Alfa Aesar, Ward Hill, MA, USA); 1-(4-trifluoromethyl)phenylpiperazine, (98%, C_11_H_13_F_3_N_2_, MW = 230.21; MP = 65–71 °C; Alfa Aesar, Ward Hill, MA, USA), 1-diphenylmethyl-piperazine (98%, C_17_H_20_N_2_, MW = 252.36, MP = 90–93°C, Fluka Ward Hill, MA, USA); 2-(piperazin-1-yl)pyrimidine (95%, C_8_H_12_N_4_, MW= 164.21; Angene, London, UK); 99% of phthalimide came from Acros Organic (Fair Lawn, NJ, USA), while 4-chloroethyl)morpholine hydrochloride (99%, MG = 186.08, MW = 184–186 °C) and 1,2,3,4-tetrahydroisoquinoline (95%, C_9_H_11_N, MW = 133.19) were purchased from Aldrich Chemical Company Inc. now part of MilliporeSigma, Burlington, MA, USA).

In order to confirm the structure of the final molecules of compounds **I**–**VI**, elemental, spectral, and mass studies were carried out. The results of the C, H, and N determinations (performed by Carlo Erba (Waltham, MA, USA) Elemental Analyzer Model NA 1500) were within ± 0.4% of the theoretical values. All melting points have not been corrected. FT-IR spectra were acquired on a Perkin-Elmer Spectrum Two, UATR FT-IR spectrometer (Perkin Elmer, Waltham, MA, USA). The samples were applied in solid form.

^1^H NMR, ^13^C NMR spectra were determined in CDCl_3_ or DMSO on a Bruker (Billerica, MA, USA) Ultra Shield 300 MHz instrument, using tetramethylsilane as internal standard (IS), and also using a Bruker AVANCE III™ 600 MHz NMR spectroscope with an Ascend™ technology magnet (active shielded superconducting magnesium, Ø 54 mm, area 14.095 T). The chemical shift values are given in ppm, with coupling constants (J) noted in Hertz (Hz) describing, respectively, s—singlet, d/dd—doublet, t—triplet, m—multiplet.

Mass spectrometry (ESI–MS). The ESI–MS experiment was performed on a CompactTM mass spectrometer (Bruker Daltonics, Bremen, Germany) equipped with a standard ESI source. The instruments operated in positive ion mode. Spectra were recorded for samples dissolved in MeOH.

#### 3.1.2. Synthesis of Compounds **I**–**VI**

For imides **I**–**IV**, **VI**, an amount corresponding to 0.0031 mol N-(2-bromoethyl)phthalimide and 0.5 g of anhydrous potassium carbonate (K_2_CO_3_) and 0.0031 mol secondary amines was suspended in 50 mL of acetonitrile (HPLC grade) and heated for 10 h at the reflux temperature of the solvent using a magnetic stirrer. The course of condensation was monitored by TLC using ethyl acetate (EA) as an eluent to develop the chromatogram (Table 2). Inorganic substances (K_2_CO_3_, KBr) were filtered hot through a paper filter, then the acetonitrile solution was completely evaporated in a vacuum, and the crude product was heated in ethanol with the addition of activated carbon (0.1 g) for preliminary purification, after filtration crystallized from ethanol. Crystals of the final products **I**–**IV**, **VI** were filtered using a Büchner funnel and then dried and subjected to basic (elemental analysis) and spectral tests (FT-IR, ^1^H NMR, ^13^C NMR, mass spectroscopy ESI-MS with fragmentation).

Imide **V** was obtained by condensing 99% phthalimide with 2-chloroethylmorpholine. Commercial 2-chloroethylmorpholine hydrochloride (0.0032 mol) was heated in an ethanolic solution of potassium ethoxylate (0.0032 mol), then filtered while hot, leaving the inorganic substance (KCl) on the filter, and the ethanol was completely evaporated in vacuo. In the next step, the crude 2-chloroethylmorpholine oil (0.003 mol) was condensed in anhydrous ethanol with phthalimide potassium salt (0.003 mol) in the presence of potassium ethoxylate (0.003 mol). The mixture was heated at the reflux of the solvent for 5 h, filtered hot through filter paper, the remaining inorganics were discarded, and the filtrate was allowed to crystallize. The obtained product was heated in ethanol with the addition of activated carbon (0.1 g) for preliminary purification, leaving the filtrate for recrystallization. The resulting crystals were filtered using a Büchner filter and air dried. The efficiency of the described method A was 47.20% of the final product. 

Method B: 0.01 mole (1.86 g) of N-(2-chloroethyl)morpholine hydrochloride was heated in a mixture of 0.01 mole of potassium ethoxylate in ethanol for 1 h. Everything was then filtered, discarding the inorganic substance (KCl), and the solvent was completely evaporated on a vacuum evaporator. The residue after evaporation in the form of a yellow oil, i.e., 0.008 mol of 2-chloroethylmorpholine and 0.008 mol of phthalimide and anhydrous potassium carbonate (micronized in a mortar) in acetonitrile, was condensed. The reaction was carried out at the reflux temperature of acetonitrile for 5 h. The mixture was then filtered hot through filter paper, discarding the K_2_CO_3_, and the filtrate was completely evaporated. Crystallization was carried out from ethanol using a small amount of activated carbon (0.1 g). The resulting crystals of the final product were filtered off using a Büchner funnel and air dried. Using this method (B), the product was obtained with a yield of 86.54%. The properties of the obtained compounds **I**–**VI** are shown in Table 2.

Below is the interpretation of the ^1^H and ^13^C NMR spectra, where the chemical shift values [ppm] of protons and carbon atoms for the final imides are indicated in relation to the remaining protons of the CDCl_3_ solvent (7.26 and 77.16 ppm, respectively). ^1^H NMR, ^13^C NMR and Mass Spectra plots are available in the Supplementary File (Figures S1–S24).

**I**: N-[2-(4-phenyl-1-piperazinyl)ethyl]-1*H*-isoindole-1,3(2*H*)-dione: [C_20_H_21_N_3_O_2_] ^1^H NMR (300 MHz, in CDCl3 [ppm]: 2.66–2.72 (distorted t-6H, -C**H**_2_-N-(C**H**_2_)_2_-, *J* = 2.1 Hz); 3.10–3.15(distorted t-4H, -(C**H**_2_)_2_-N-C_6_H_5_, *J* = 2.4 Hz); 3.84–3.89 (t-2H, =N-C**H**_2_-CH_2_-, *J* = 2.2 Hz); 6.80–6.90 (m-3H, aromatic H at C_2_, C_4_,C_6_ of phenyl, *J* = 11, 3, 2 Hz); 7.20–7.28 (m-2H, aromatic H of C_3_, C_5_ of phenyl, *J* = 3, 2, 0.5 Hz); 7.70–7.75 (t-2H, aromatic H at C_3_ and C_6_ of phthalimide, *J* = 3, 2, 0.5 Hz); 7.80–7.90 (t-2H, H aromatic at C_4_ and C_5_ of phthalimide, *J* = 1.1 Hz); ^13^C NMR (600 MHz, in CDCl_3_) [ppm] 42.06 (1C), 50.67 (2C), 53.47(1C), 55.35(2C), 118.20 (1C), 120.98 (1C), 123.01 (2C), 123.36 (2C),133.99 (1C), 141.11 (1C), 152.23 (2C), 168.54 (2x C=O); MS-MS [ppm] = [L+H]^+^ 336.1707, found 336.1765 (17.25), [L+Na]^+^ 358.1526, found 358.1591 (18.15), [L+H+CH_3_OH]^+^ 368.1969, found 368.2039 (19.01); elemental analysis [%] C, H, N = 71.62, 6.31, 12.53, found 71.65, 6.27, 12.59 ( 0.03, 0.04, 0.06 %, appropriately). Spectra are included in the Supplementary Materials: Figures S1–S4 (^1^H NMR, ^13^C NMR, Mass spectra and Mass spectra of fragmentation for I).

**II**: N-{2-[4-(4-trifluoromethylphenyl)-1-piperazinyl]ethyl}-1*H*-isoindole-1,3(2*H*)-dione [C_21_H_20_F_3_N_3_O_2_]: ^1^H NMR (300 MHz, in CDCl3 [ppm]: 2.65–2.76 (distorted t-6H, -C**H**_2_-N-(C**H**_2_)_2_-, *J =* 1.3, 1.1, 0.5 Hz); 3.15–3.25 (distorted t-4H, -(C**H**_2_)_2_-N-C_6_H_4_CF_3_, *J* = 1.5 Hz); 3.85–3.90 (t-2H, =N-C**H**_2_-CH_2_-, *J* = 2.1 Hz); 6.85–6.95 (dd-2H, aromatic H at C_4_,C_6_ of phenyl, *J* = 1.3, 1.1, 0.5 Hz); 7.40–7.50 (m-2H, aromatic H of C_3_, C_5_ of phenyl); 7.70–7.75 (m-2H, aromatic H at C_3_ and C_6_ of phthalimide); 7.80–7.90 (dd-2H, H aromatic at C_4_ and C_5_ of phthalimide *J* = 1.1 Hz. ^13^C NMR (60MHz, in CDCl_3_) [ppm] 49.28 (1C), 50.49 (2C), 59.74(3C), 116.34 (1C), 119.96 (1C), 125.54 (2C), 129.09 (2C), 131.00 (1C), 139.18 (1C), 151.16 (1C), 167.31 (2x C=O); MS-MS [ppm] = [L+H]^+^ 404.1580, found 404.1636 (13.86), [L+Na]^+^ 426.1400, found 426.1457 (13.38), [L+H+CH_3_OH]^+^ 436.1843, found 436.1905 (14.21); elemental analysis [%] C, H, N = 62.53, 4.99, 10.42, found 62.67, 4.31, 10.23 (0.14, 0.32, 0.19%, appropriately). Spectra are included in the Supplementary Materials: Figures S5–S8 (^1^H NMR, ^13^C NMR, Mass spectra and Mass spectra of fragmentation for II).

**III**: N-{2-[4-(4-diphenylomethylo)-1-piperazinyl]ethyl}-1*H*-isoindole-1,3(2*H*)-dione [C_27_H_27_N_3_O_2_] ^1^H NMR (300 MHz, in CDCl3 [ppm]: 2.30–2.70 (m-10H, -C**H**_2_-N-(C**H**_2_)_2_-) (dd *J* = 19.2, 6.7, 2.3 Hz)+ -(C**H**_2_)_2_-N-CH=(C_6_H_5_)_2_(t, *J* = 6.5 Hz); 3.75–3.85 (t-2H, =N-C**H**_2_-CH_2_-) *J* = 2.1 Hz; 4.10–4.20 (s-1H, -N-C**H**=(C_6_H_5_)_2_), 7.10–7.19 (dd-4H, aromatic H at C_5_,C_3_ of phenyl)(*J* = 2.4 Hz); 7.20–7.30 (t-2H, aromatic H of C_4_ of phenyl; *J* = 11.1, 2.4 Hz); 7.35–7.45 (t-2H, aromatic H of phenyl, *J* = 2.2 Hz); 7.65–7.75 (t-2H, H aromatic at C_3_ and C_6_ of phthalimide, *J* = 2.2 Hz); 7.80–7.90 (m-2H, aromatic H at C_4_ and C_5_ of phtalimide). ^13^C NMR (600 MHz, in CDCl_3_) [ppm] 48.74 (1C), 53.07 (2C), 61.56 (2C), 65.20(1C), 68.66 (1C), 112.24 (2C), 116.01 (2C), 122.47–123.50 (2C), 126.08 (2C), 129.53 (2C), 131.68 (2C), 132.03 (2C), 134.18 (2C), 151.22 (2C), 168.57 (C=O), 168.18 (C=O); MS-MS [ppm] = [L+H]^+^ 426.2176, found 426.2235 (13.84), [L + Na]^+^ 448.1995, found 448.2061 (14.73), [L+H+CH_3_OH]^+^ 458.2438, found 458.2513 (16.37). Spectra are included in the Supplementary Materials: Figures S9–S12 (^1^H NMR, ^13^C NMR, Mass spectra and Mass spectra of fragmentation for III).

**IV**: N-{2-[4-(4-pyrimidyn-2-ylo-1-piperazinyl]ethyl}-1*H*-isoindole-1,3(2*H*)-dione [C_18_H_19_N_5_O_2_] ^1^H NMR (600 MHz, in CDCl3 [ppm]: 2.63–2.74 (t-6H, -C**H**_2_-N-(C**H**_2_)_2_; 3.80–3.85 (t-H, -(CH_2_)_2_-N-C_4_H_3_N_2_, *J* = 6.7 Hz), 3.91-3.95 (t-2H, =N-C**H**_2_-CH_2_-, *J* = 6.5 Hz); 6.50 (s-1H, H aromatic at C_5_ of pyrimidine), 7.73–7.88 (t-4H, aromatic H of phtalimide, *J* = 1.3 Hz); 8.31–8.35 (t-2H, aromatic H at C_4_ and C_5_ of pyrimidine, *J =* 1.1 Hz). ^13^C NMR (600 MHz, in CDCl_3_) [ppm] 35.07, 43.51, 52.83, 55.71 (C of methylene groups of alkyl and piperazine); 109.91, 123.30, 132.17, 133.97, 157.73, 161.57 (6C of phtalimide and 4C of pyrimidine); 168.38 (2x C=O).

MS-MS [ppm] = [L+H]^+^ 338.1612, found 338.1604 (2.37), [L+Na]^+^ 360.1431, found 360.1414 (4.72), [L+H+CH_3_OH]^+^ 370.1874, found 370.1865 (2.43), [L + K] 376.1170 found 376.1157 (3.46). Spectra are included in the Supplementary Materials: Figures S13–S16 (^1^H NMR, ^13^C NMR, Mass spectra and Mass spectra of fragmentation for IV).

**V**: N-(2-morpholine)ethyl-1*H*-isoindole-1,3(2*H*)-dione [C_14_H_16_N_2_O_3_] ^1^H NMR (600 MHz, in CDCl3) [ppm]: 2.58–2.70 (t-6H, -C**H**_2_-N-(C**H**_2_)_2_-; *J* = 2.2 Hz); 3.69–3.75 (t-4H, -(CH_2_)_2_-O, *J* = 1.8 Hz), 3.87-3.95 (t-2H, =N-C**H**_2_-CH_2_-, *J* = 1.1 Hz); 7.74–7.88 (t-4H, aromatic H of phtalimide, *J* = 1.1 Hz); ^13^C NMR (600MHz, in CDCl_3_) [ppm] 53.40 (1C), 56.08 (1C), 66.77(2C) 77.26 (2C), 123.28 (2C), 132.18 (2C), 133.97 (2C), 168.38 (2x C=O).

MS-MS [ppm] = [L+H]^+^ 261.1234, found 261.1231 (1.15), [L+Na]^+^ 283.1053, found 283.1042 (3.89), [L+H+CH_3_OH]^+^ 293.1496, found 293.1489 (2.39). Spectra are included in the Supplementary Materials: Figures S17–S20 (^1^H NMR, ^13^C NMR, Mass spectra method A and Mass spectra method B for V).

**VI**: N-[2-(3,4-dihydro-1*H*-isoquinolin-yl)ethyl-1*H*-isoindole-1,3(2*H*)-dione [C_19_H_18_N_2_O_2_] ^1^H NMR (600 MHz), in CDCl3 [ppm]: 2.69–3.23 (t-6H, -C**H**_2_-N-(C**H**_2_)_2_-, *J* = 16 Hz); 3.81–3.83 (t-2H, -CH_2_-C**H**_2_-C_6_H_4_, *J*= 2Hz), 3.95–3.99 (t-2H, =N-C**H**_2_-CH_2_-, *J* = 4 Hz); 7.05–7.28 (t-4H, H aromatic at isoquinoline), 7.71–7.72 (t-2H, aromatic H of phtalimide, *J* = 1.1 Hz); 7.84–7.86 (t-2H, aromatic H of phtalimide, *J* = 2 Hz); ^13^C NMR (600MHz, in CDCl_3_) [ppm] 28.35 (1C), 35.03 (1C), 50.02 (1C), 54.74(2C), 123.30 (2C), 126.65 (2C), 132.16 (2C), 133.94 (2C), 168.40 (2x C=O). MS-MS [ppm] = [L+H]^+^ 307.1441, found 307.1447 (1.95), [L+Na]^+^ 329.1260, found 329.1255 (1.52), [L+H+CH_3_OH]^+^ 339.1703, found 339.1709 (1.77), [L+K]^+^ 345.1000, found 345.0996 (1.16). Spectra are included in the Supplementary Materials: Figures S21–S24 (^1^H NMR, ^13^C NMR, Mass spectra and Mass spectra of fragmentation for VI).

### 3.2. In Silico Studies

For molecular docking and molecular dynamics studies, the following crystal structures from the RCSB database were used: 1EVE [26] and 1P0I [29]. The Gaussian 2016 C.01 software package [35] was used to optimize the 3D structure of the tested derivatives. The B3LYP/6-31+G (d.p) basic set was applied. AutoDock Tools 1.5.6 [36] was used to prepare input files for docking. For imides, rotatable bonds were attributed, nonpolar hydrogens were merged, and partial charges were added. For the AChE and BuChE, co-crystallized molecules and water were removed (except three water molecules in the AChE pocket). Kollman partial charges and non-polar hydrogens were added. The grid box was set according to the enzyme’s gorge. The simulation was performed using the AutoDockVina 1.1.2 script [37]. The docking protocol was first validated by self-docking the co-crystallized ligand.

The molecular dynamic simulation was carried out using Gromacs 2021.2 software [38], using the CHARMM36m force field [39]. The ligand topology was prepared using the CHARMM-GUI server [40,41]. The TP water model KCl (0.15M) for neutralization was applied. The energy of the system was minimized and then the system was equilibrated for 125 ps. Next, MD simulation was performed for 100 ns with a step of 2 fs, considering a constant pressure of 1 bar and constant temperature of 303 K. The binding free energy ΔG_bind_ was calculated using the gmx_MMPBSA tool v.1.6.1 [28]. The visualizations of the in silico studies results were created using ChimeraX 1.7 [42] software and LigPlot + v.2.2 software [43]. The RMSD plots were made using OriginPro 2024 v.10.1.

The toxicity properties and probability of penetration of the BBB were calculated using ProTox 3.0 tool [44].

### 3.3. In Vitro Studies

The following reagents were used to evaluate the inhibiting activity: acetylcholinesterase (AChE) from electrophorus electricus (Sigma-Aldrich, Saint Louis, MO, USA), butyrylcholinesterase (BuChE) from horse serum, acetylthiocholine iodide (Sigma-Aldrich, Saint Louis, MO, USA), butyrylthiocholine iodide (Sigma-Aldrich, Saint Louis, MO, USA), 5,5′-Dithiobis-(2-nitrobenzoic acid) (DTNB, Sigma-Aldrich, Saint Louis, MO, USA). The enzymes were prepared by dissolving in phosphate buffer, pH = 8.0) with the final concentration 2 U/mL for AChE and 4 U/mL for BuChE. The stock solutions of acetylthiocholine iodide (10^−3^ mol/dm^3^), butyrylthiocholine iodide (10^−3^ mol/dm^3^) and DTNB (10^−3^ mol/dm^3^) were prepared in phosphate buffer. The inhibitory activity of derivatives was evaluated by Ellman’s colorimetric method in a 96-well plate. First, the enzyme solution with the tested compound was incubated for 20 min at 37 °C. Next, acetylthiocholine iodide or butyrylthiocholine iodide and DTNB were added. The absorbance of each plate was measured for the inhibitor’s concentration 10^−4^–10^−9^ mol/dm^3^ and for a blank sample, and donepezil (Sigma-Aldrich, Saint Louis, MO, USA) (measurement was replicated three times) at 412 nm using a microplate reader (Elisa Multiskan GO, Thermo Scientific Waltham, MA, USA). The IC_50_ values were calculated by nonlinear regression (dose–response curve) using OriginPro 2024. 

## 4. Conclusions

Two in silico methods, i.e., molecular docking and molecular dynamics, made it possible to propose six new isoindole-1,3-dione derivatives as potential AChE and BuChE inhibitors. The in vitro study confirmed the results obtained from in silico studies. All tested imides showed inhibitory activity against AChE and BuChE (except imide V). The IC_50_ values indicate the high potential of derivatives containing appropriately substituted arylpiperazine residues. In addition to interaction with the CAS region, interaction with the PAS region plays an important role in the design of potential inhibitors. The tested compounds are a good base for our subsequent studies. We plan a structural modification, including additional substituents in the phenyl ring or modification of the linker length, as well as the introduction of a substituent in the phthalimide aryl ring. Also of interest is the anti-inflammatory activity of similar imides [24]. It would be desirable for the new compounds to have simultaneous anti-inflammatory and neuroprotective effects by inhibiting various inflammatory signaling pathways, including cyclooxygenases, and effectively inhibiting cholinesterases.

## Figures and Tables

**Table 1:** The binding affinity and binding free energy for tested compounds—results from molecular docking and molecular dynamics simulation.

Compound	Binding Affinity (Docking) (kcal/mol)		Binding Free Energy (MD) (kcal/mol)	
	ACHE	BuCHE	ACHE	BuCHE
**I**	−9.7	−9.9	−12.87 ± 1.66	−13.78 ± 1.42
**II**	−9.3	−9.9	−15.15 ± 1.91	−13.94 ± 1.71
**III**	−10.1	−11.0	−15.71 ± 2.37	−15.41 ± 1.81
**IV**	−9.2	−9.4	−7.08 ± 1.43	−7.18 ± 1.37
**V**	−8.2	−8.3	−7.35 ± 1.74	−1.91 ± 0.51
**VI**	−10.2	−10.2	−14.34 ± 2.02	−7.03 ± 0.94
Donepezil	−10.9	−10.0	-	-

**Table 2:** The properties of obtained compounds **I**–**VI**.

Comp.	Formula	m.p. (°C) Ethanol	Yield (%)	TLC Rf Ethyl Acetate (EA)	FT-IR (cm^−1^) C=O Arene -CH_2_-
**I**	C_20_H_21_N_3_O_2_ 335.4	156–158	78.85	0.87	1715, 1775 695, 710
**II**	C_21_H_20_F_3_N_3_O_2_ 403.40	137–139	56.6	0.74	1720, 1770 700, 715
**III**	C_27_H_27_N_3_O_2_ 425.52	150–152	75.7	0.84	1710, 1770 710
**IV**	C_18_H_19_N_5_O_2_ 337.40	113–115	49.79	0.80	1710, 1780 710
**V**	C_14_H_16_N_2_O_3_ 260.29	134–136	47.20 (A) 86.54 (B)	0.78	1700, 1780 3030, 2850
**VI**	C_19_H_18_N_2_O_2_ 306.40	128–130	55.48	0.85	1710, 1780 735 3030, 2850

(A)—Method A, (B)—Method B.

**Table 3:** Inhibitory activity of imides **I**–**VI** against AChE and BuChE.

Compound	IC_50_ [μM]		Selectivity	
	AChE	BuChE	AChE	BuChE
**I**	1.12 ± 0.30	24.67 ± 1.74	22.02	0.05
**II**	2.31 ± 0.36	24.71 ± 3.78	10.70	0.09
**III**	1.94 ± 0.43	21.24 ± 4.84	10.95	0.09
**IV**	3.90 ± 0.43	68.88 ± 7.82	17.66	0.06
**V**	16.20 ± 2.37	>1000		
**VI**	1.14 ± 0.17	52.11 ± 9.73	45.71	0.02
Donepezil	0.04 ± 0.01	3.29 ± 0.85	82.55	0.01

**Table 4:** Predicted toxicity properties and probability of penetration of the blood–brain barrier for the studies imides.

	Compound					
	I	II	III	IV	V	VI
Carcinogenicity	inactive	inactive	inactive	inactive	inactive	inactive
Immunotoxicity	inactive	inactive	inactive	inactive	inactive	inactive
Mutagenicity	inactive	inactive	inactive	inactive	inactive	inactive
Cytotoxicity	inactive	inactive	inactive	inactive	inactive	inactive
LD_50_ (mg/kg)	4000	728	4000	4000	4000	315
class	5	4	5	5	5	4
BBB (probability)	0.98	0.98	0.98	0.99	0.92	0.98

## Data Availability

The data are available on request from the corresponding authors.

## Supplementary Materials

The following supporting information can be downloaded at: https://www.mdpi.com/article/10.3390/molecules29153528/s1, Figure S1: ^1^H NMR spectrum of the compound I; Figure S2: ^13^C NMR spectrum of the compound I; Figure S3: Mass spectrum of the compound I; Figure S4: Mass spectrum of fragmentation the compound I; Figure S5: ^1^H NMR spectrum of the compound II; Figure S6: ^13^C NMR spectrum of the compound II; Figure S7: Mass spectrum of the compound II; Figure S8: Mass spectrum of fragmentation the compound II; Figure S9: ^1^H NMR spectrum of the compound III; Figure S10: ^13^C NMR spectrum of the compound III; Figure S11: Mass spectrum of the compound III; Figure S12: Mass spectrum of fragmentation the compound III; Figure S13: ^1^H NMR spectrum of the compound IV; Figure S14: ^13^C NMR spectrum of the compound IV; Figure S15: Mass spectrum of the compound IV; Figure S16: Mass spectrum of fragmentation the compound IV; Figure S17: ^1^H NMR spectrum of the compound V; Figure S18: ^13^C NMR spectrum of the compound V; Figure S19: Mass spectrum of the compound V; Figure S20: Mass spectrum of fragmentation the compound V; Figure S21: ^1^H NMR spectrum of the compound VI; Figure S22: ^13^C NMR spectrum of the compound VI; Figure S23: Mass spectrum of the compound VI; Figure S24: Mass spectrum of fragmentation the compound VI.
