# Supplementary material for: A Series of Novel 1-H-isoindole-1,3(2H)-dione Derivatives as Acetylcholinesterase and Butyrylcholinesterase Inhibitors: In Silico, Synthesis and In Vitro Studies
Source: Molecules. 2024 Jul 26;29(15):3528. doi: 10.3390/molecules29153528 (PMC11313788; doi:10.3390/molecules29153528)
Supplement: Supplementary file 1 [file molecules-29-03528-s001.zip › molecules-3116727-supplementary.pdf]

Supplementary Information for

# A series of novel 1-*H*-isoindole-1,3(2*H*)-dione Derivatives as Acetylcholinesterase and Butyrylcholinesterase Inhibitors: In Silico, Synthesis and In Vitro Studies

**Edward Krzyżak [1]*, Aleksandra Marciniak [1], Dominika Szkatuła [2], Klaudia A. Jankowska [3], Natalia Dobies [3] and Aleksandra Kotynia [1]**

[1] Department of Basic Chemical Sciences, Faculty of Pharmacy, Wroclaw Medical University, Borowska 211a, 50-556 Wrocław, Poland; aleksandra.marciniak@umw.edu.pl (A.M.); aleksandra.kotynia@umw.edu.pl (A.K.)
[2] Department of Medicinal Chemistry, Wroclaw Medical University, Borowska 211, 50-556 Wroclaw, Poland; dominika.szkatula@umw.edu.pl; (D.S.)
[3] Student Scientific Club of Medicinal Chemistry, Wroclaw Medical University, Borowska 211, 50-556 Wroclaw, Poland
* Correspondence: edward.krzyzak@umw.edu.pl; Tel.: +48-71-784-03-29

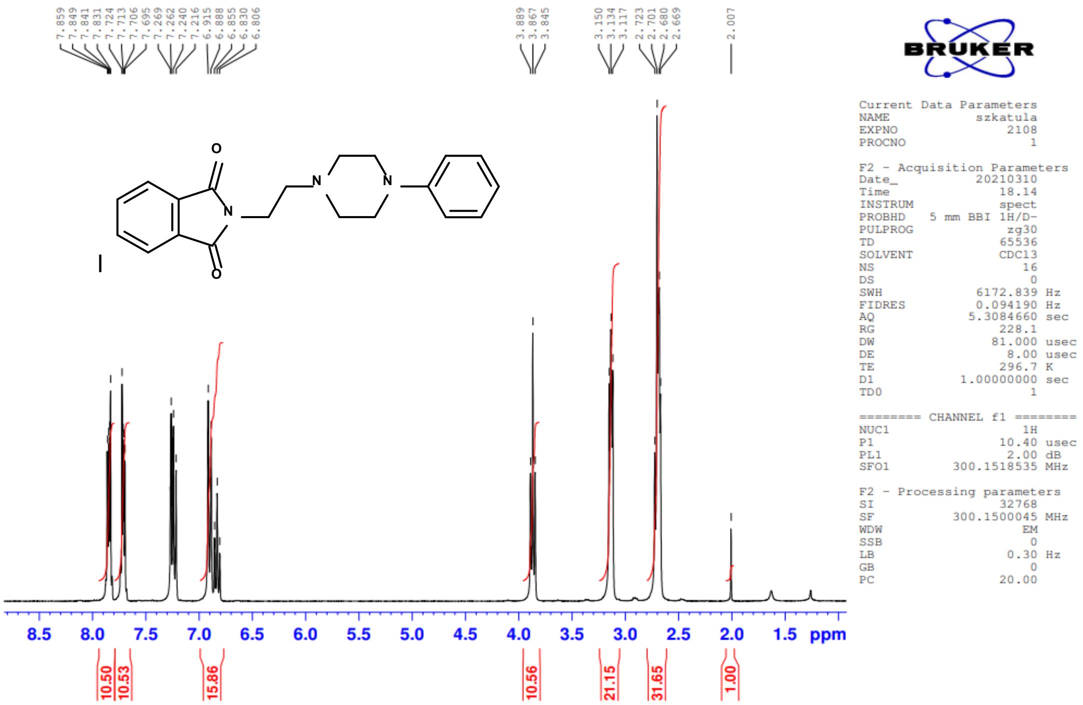


Figure S1: $^{1}$H NMR spectrum of the compound I

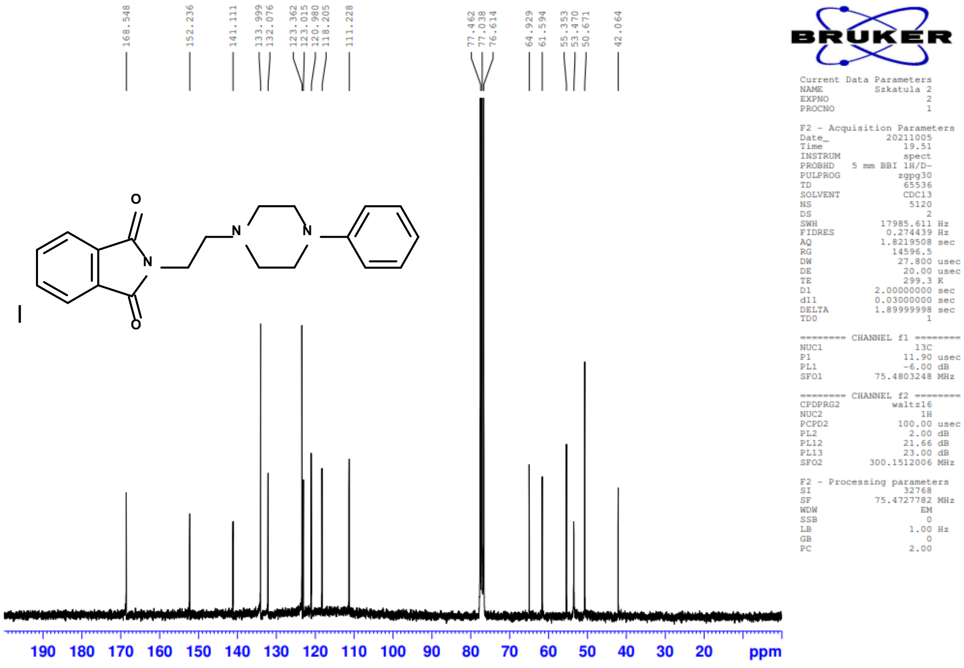

Figure S2: $^{13}$C NMR spectrum of the compound I

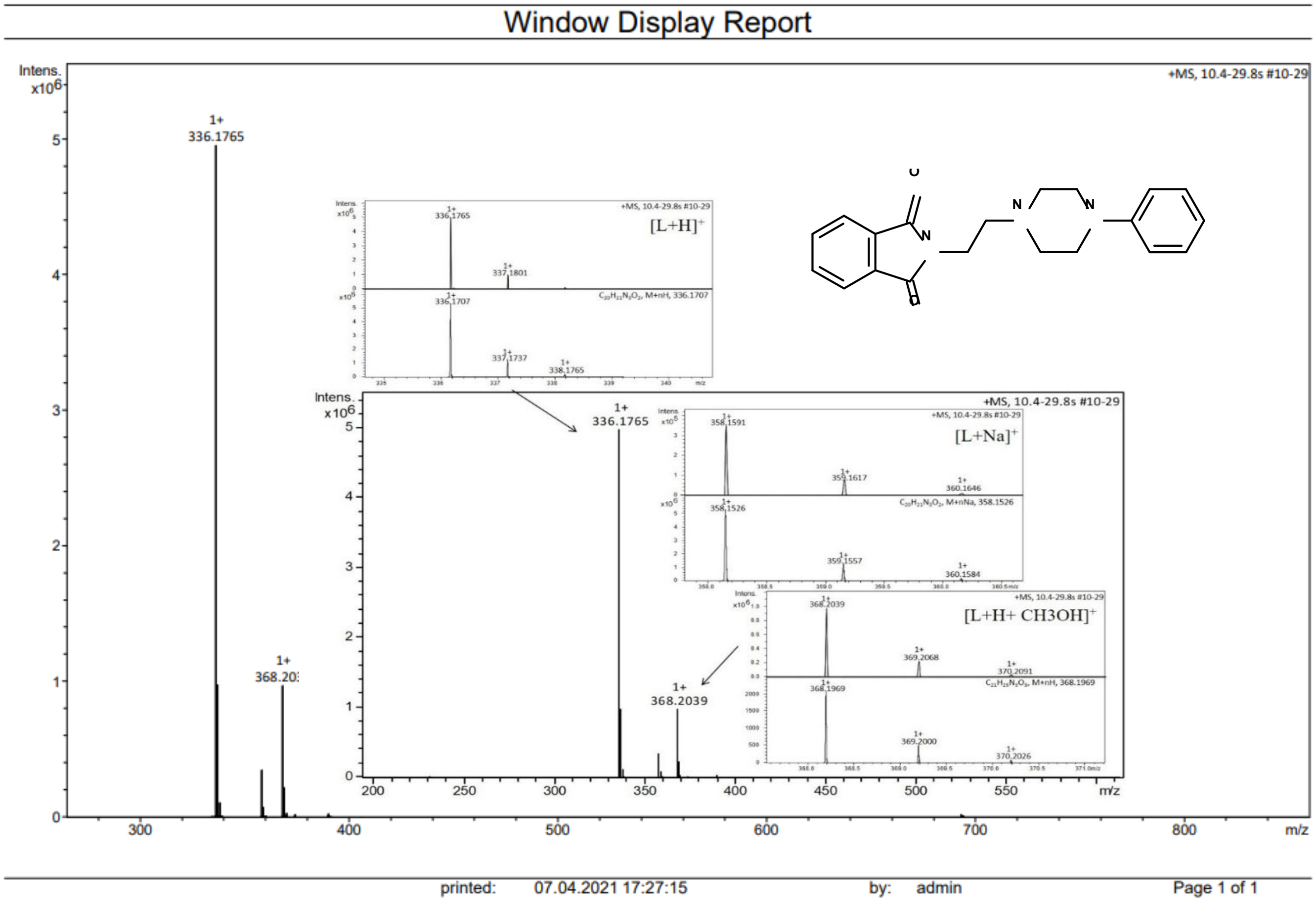


Figure S3: Mass spectrum of the compound I

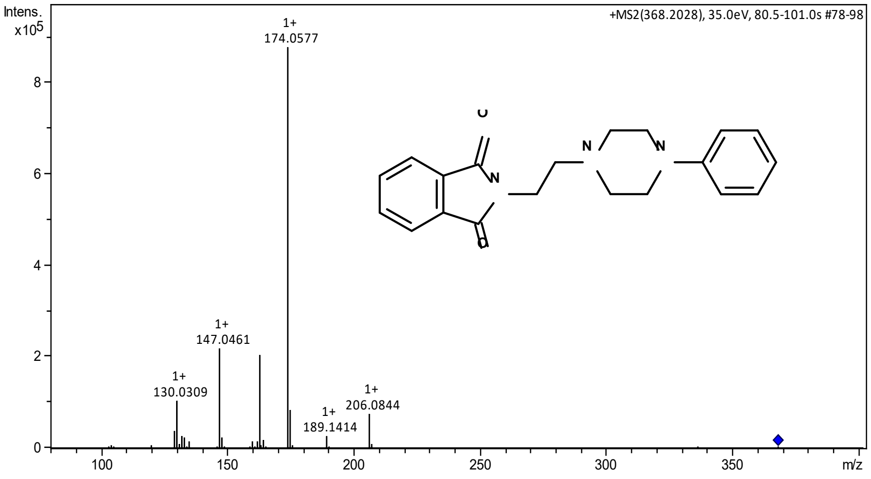


Figure S4: Mass spectrum of fragmentation the compound I

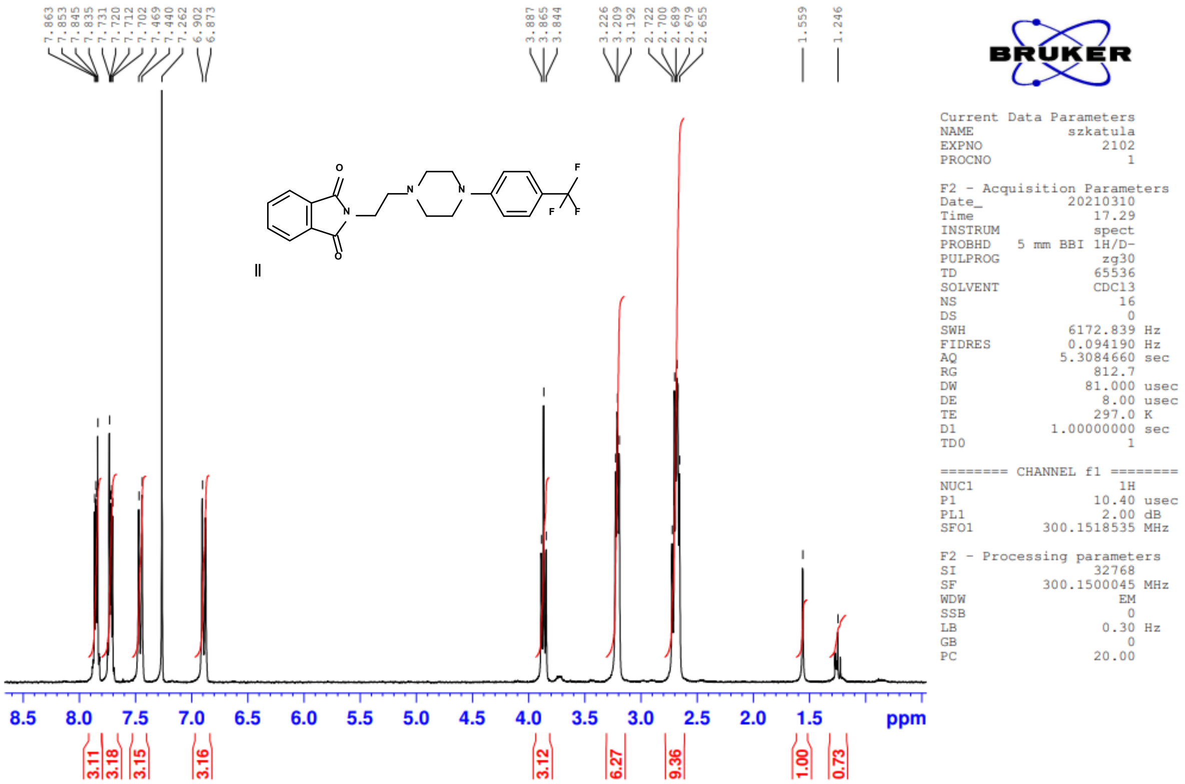


Figure S5: $^{1}H$ NMR spectrum of the compound II

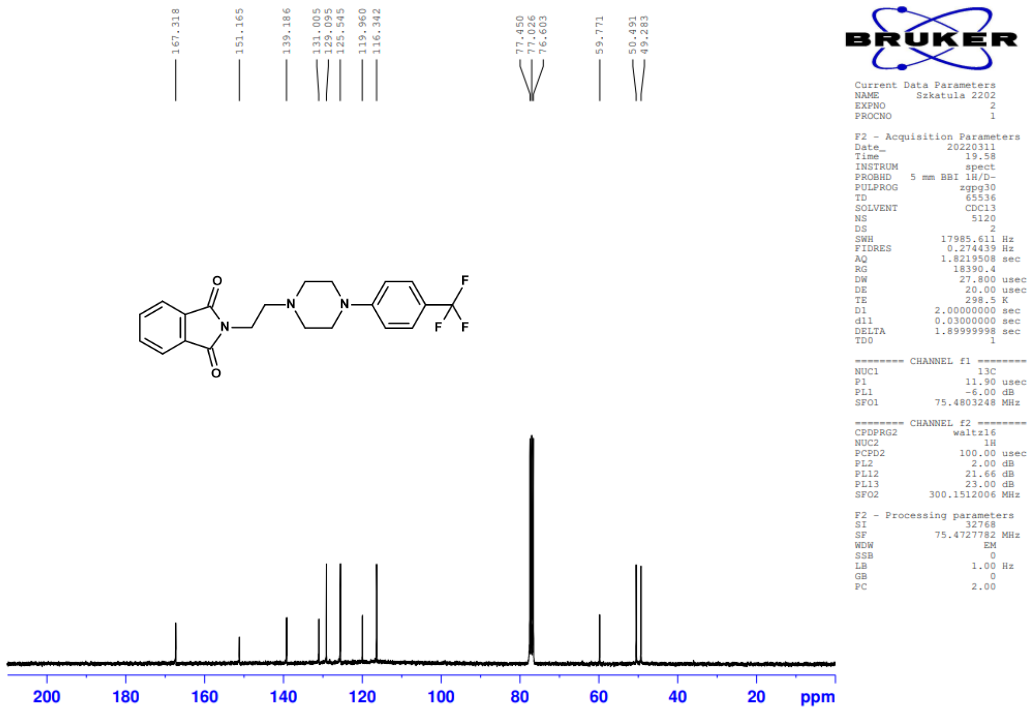

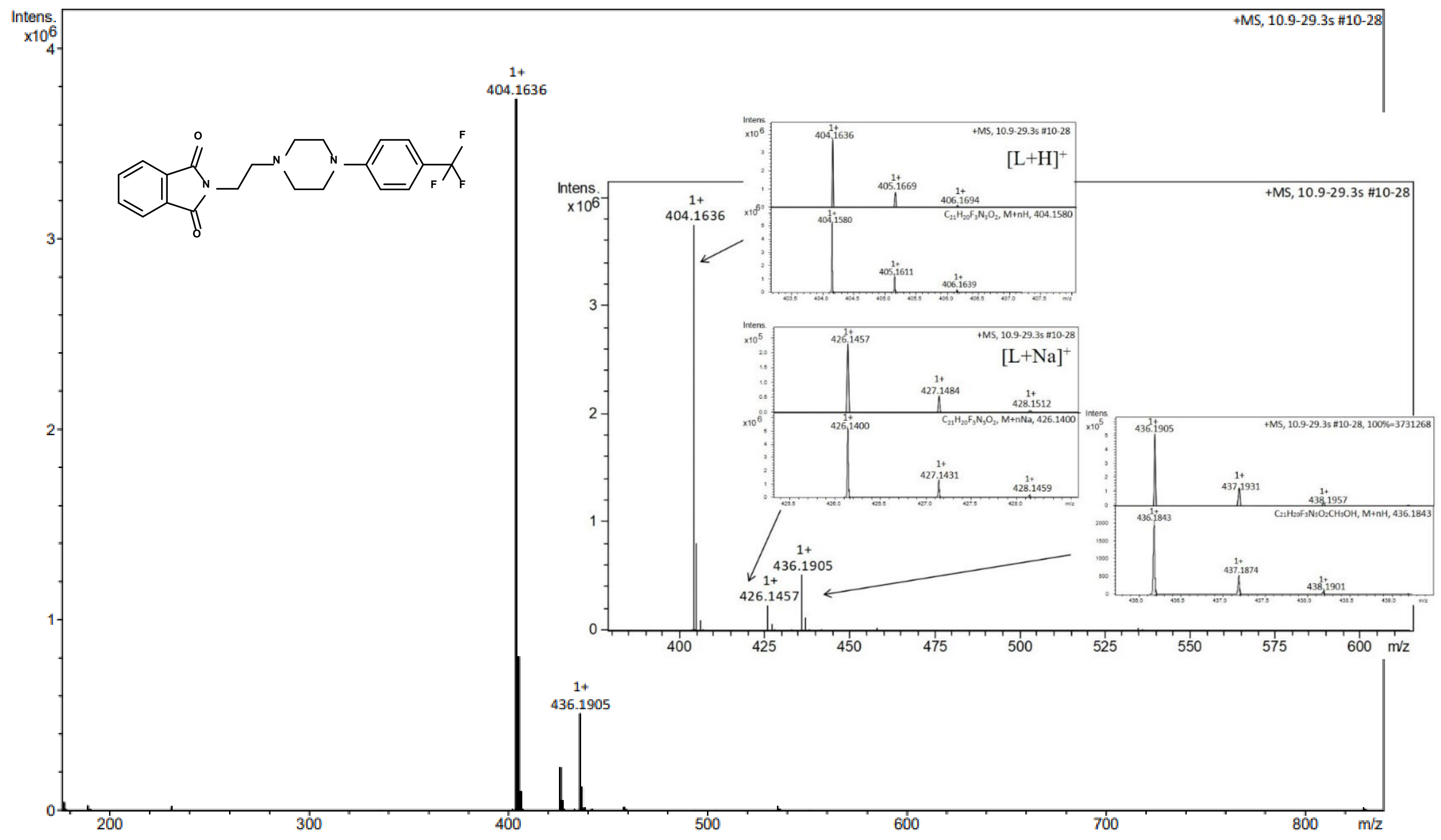




Figure S7: Mass spectrum of the compound II

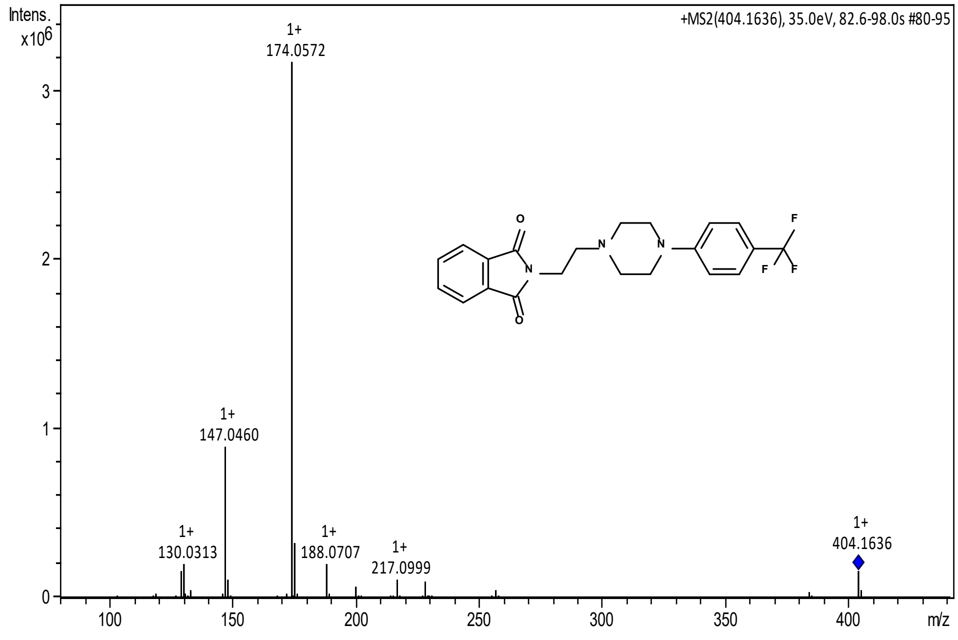

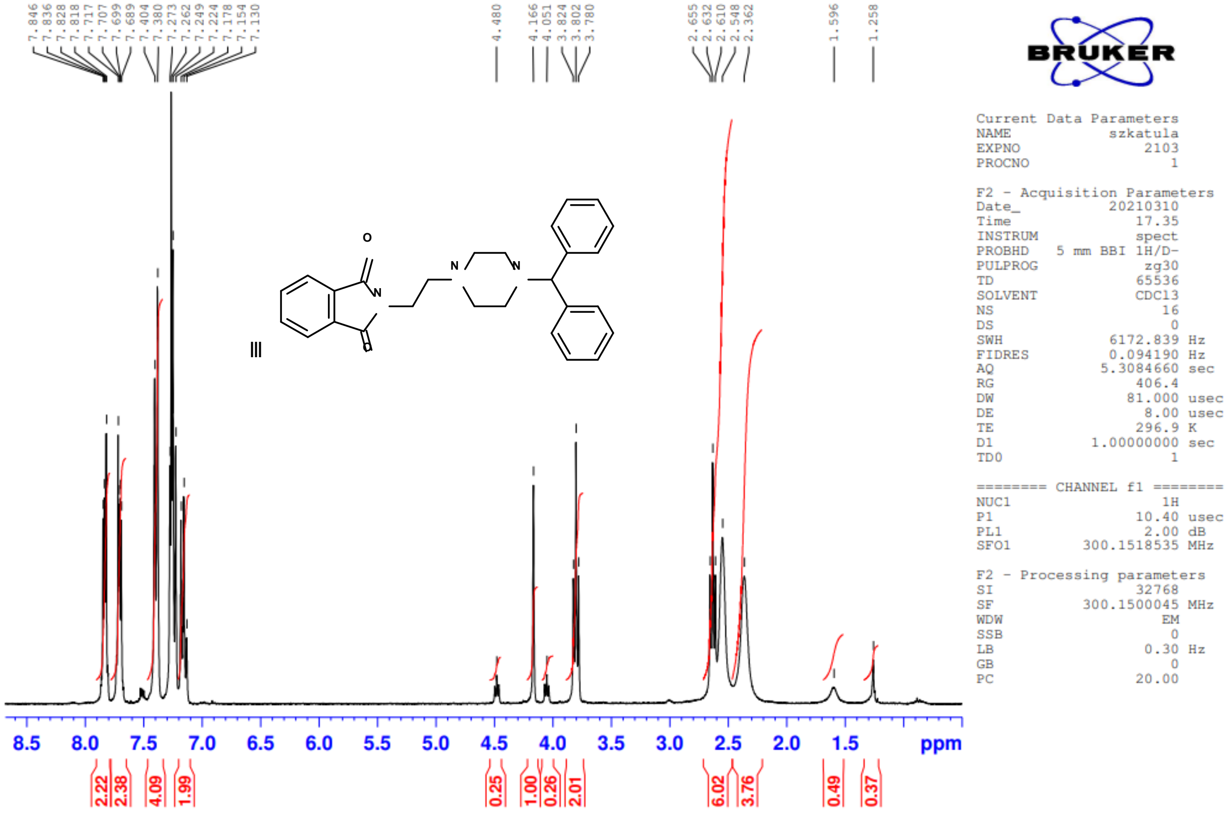


Figure S9: $^{1}$H NMR spectrum of the compound III

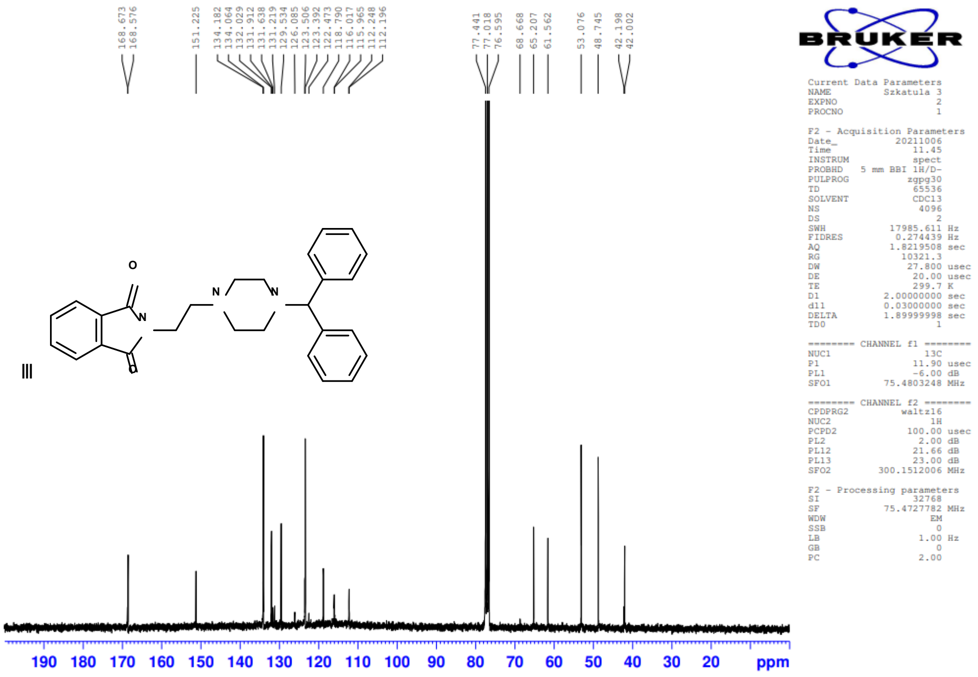

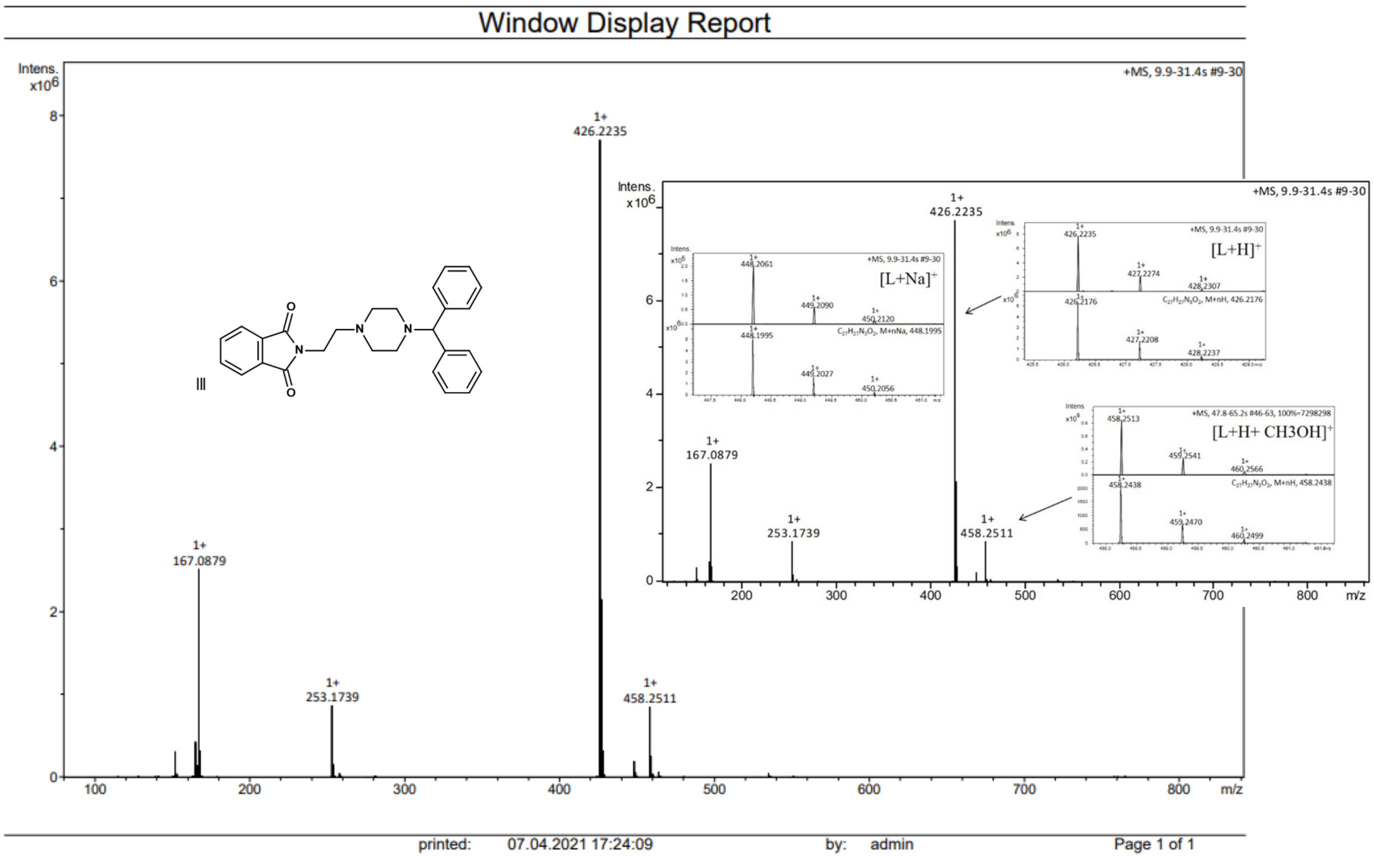


Figure S11: Mass spectrum of the compound III

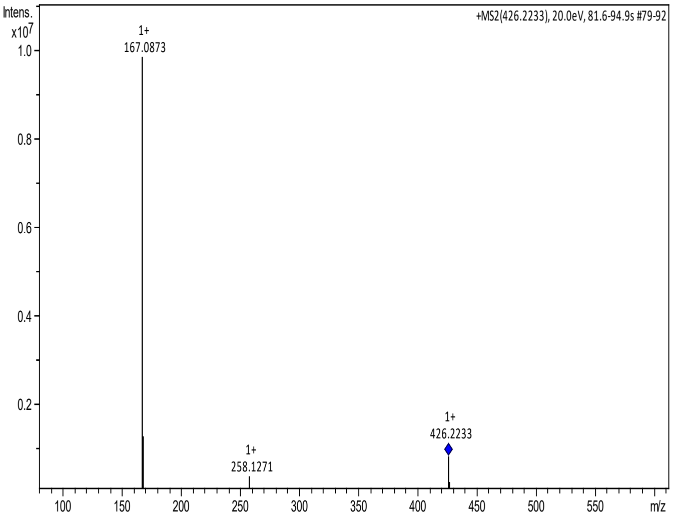


Figure S12: Mass spectrum of fragmentation of the compound III

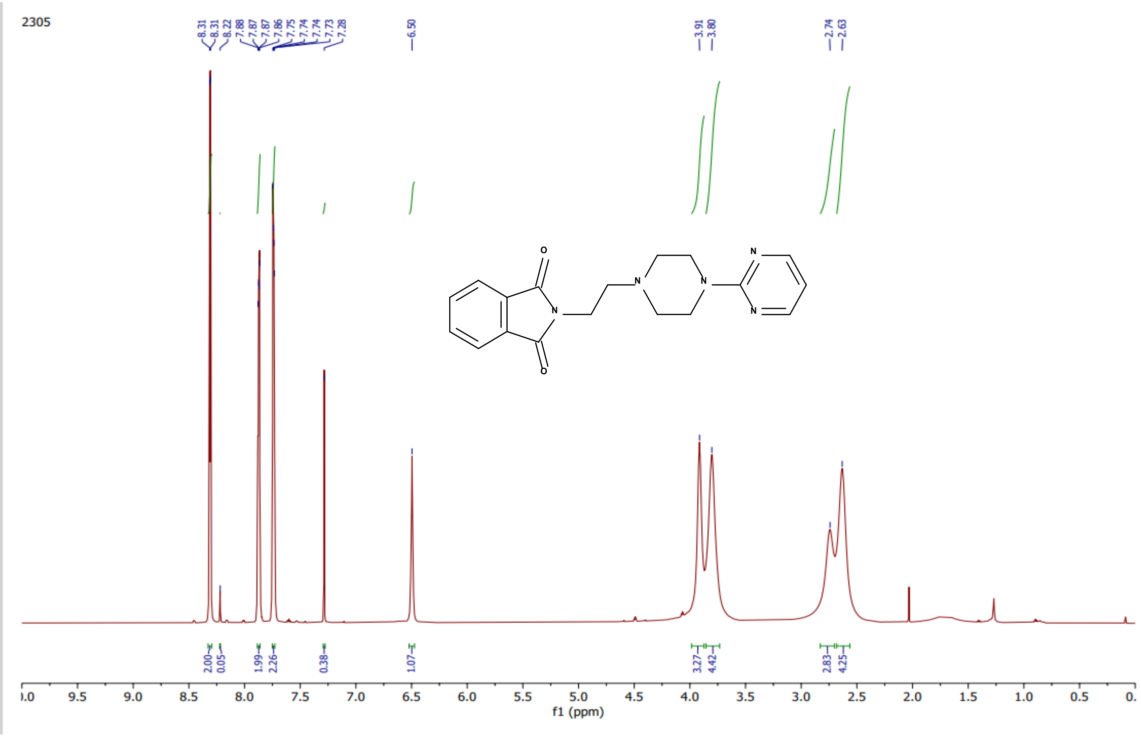


Figure S13: $^{1}$H NMR spectrum of the compound IV

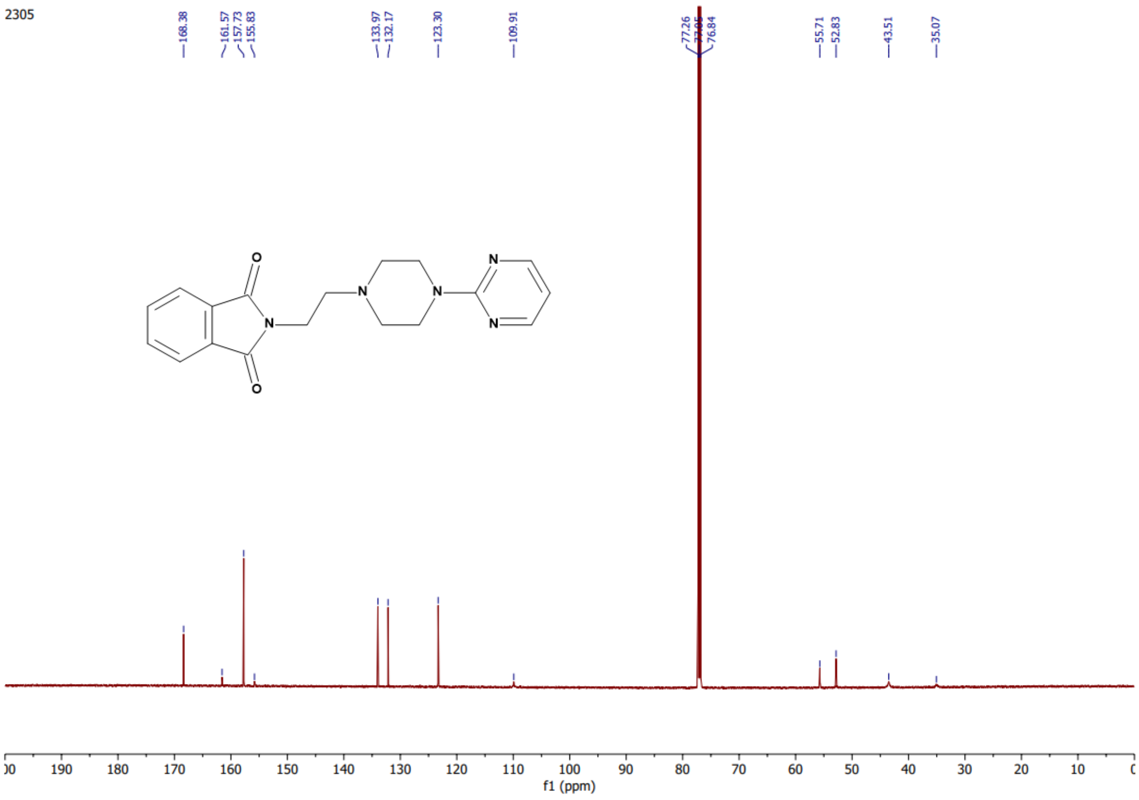

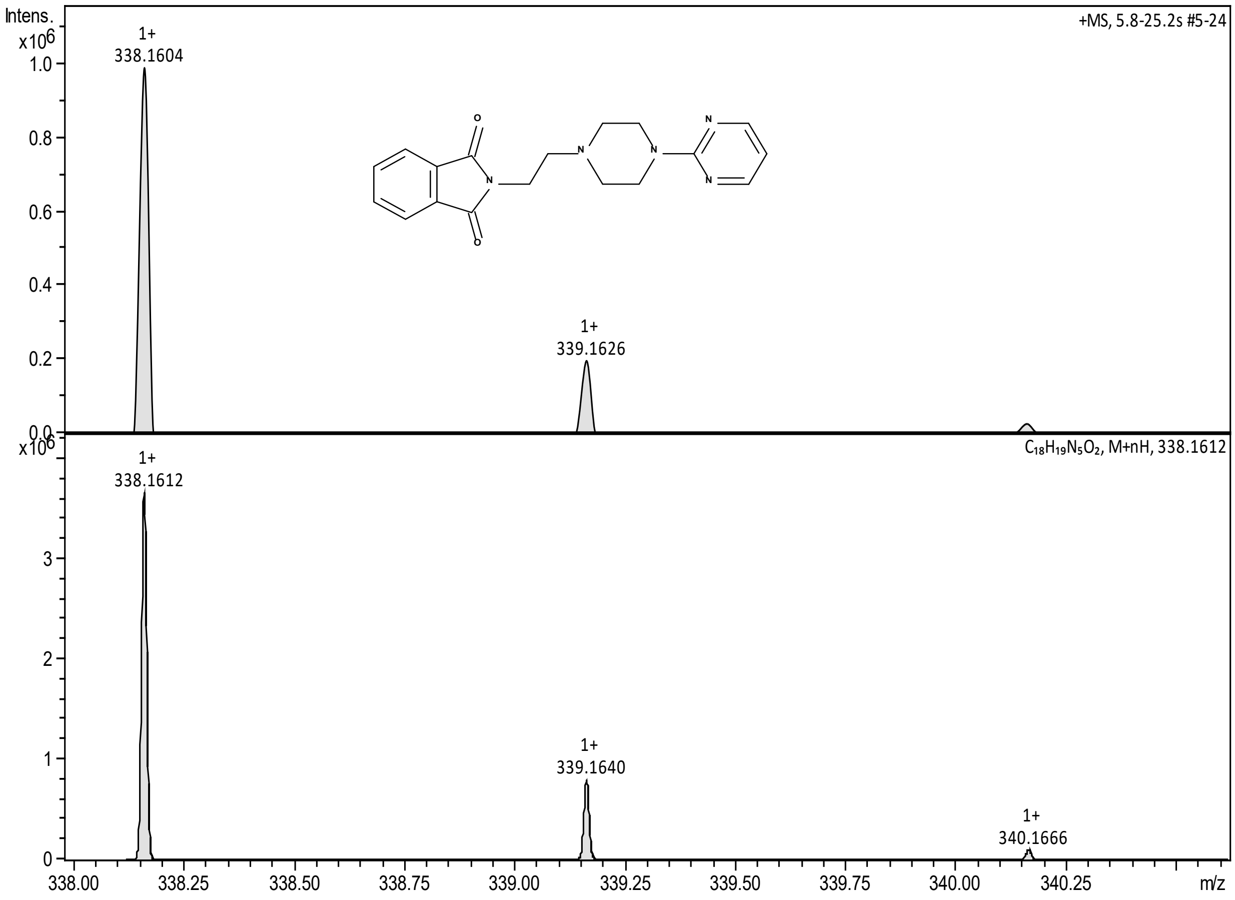


Figure S15: Mass spectrum of the compound IV

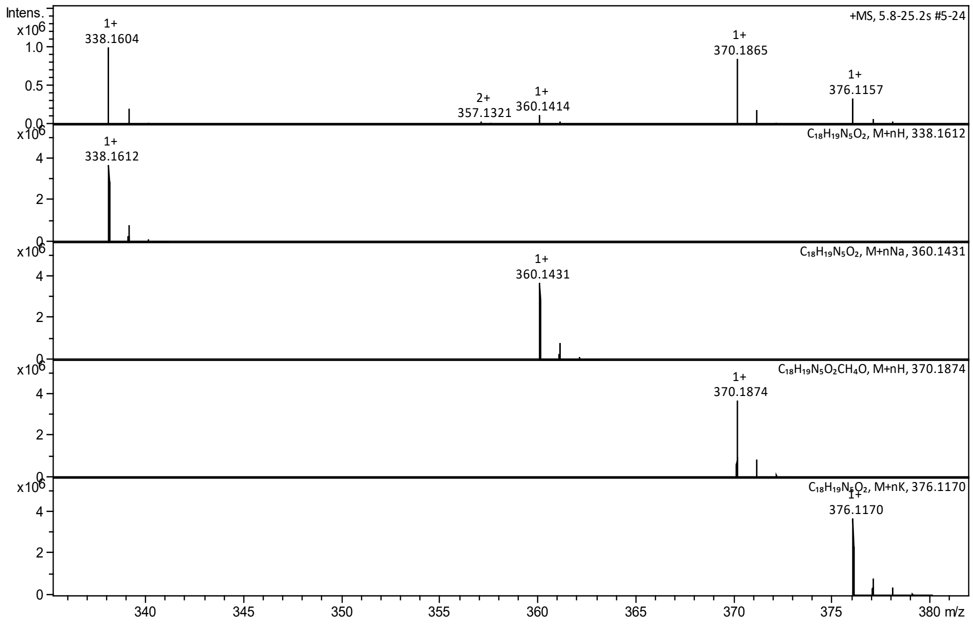

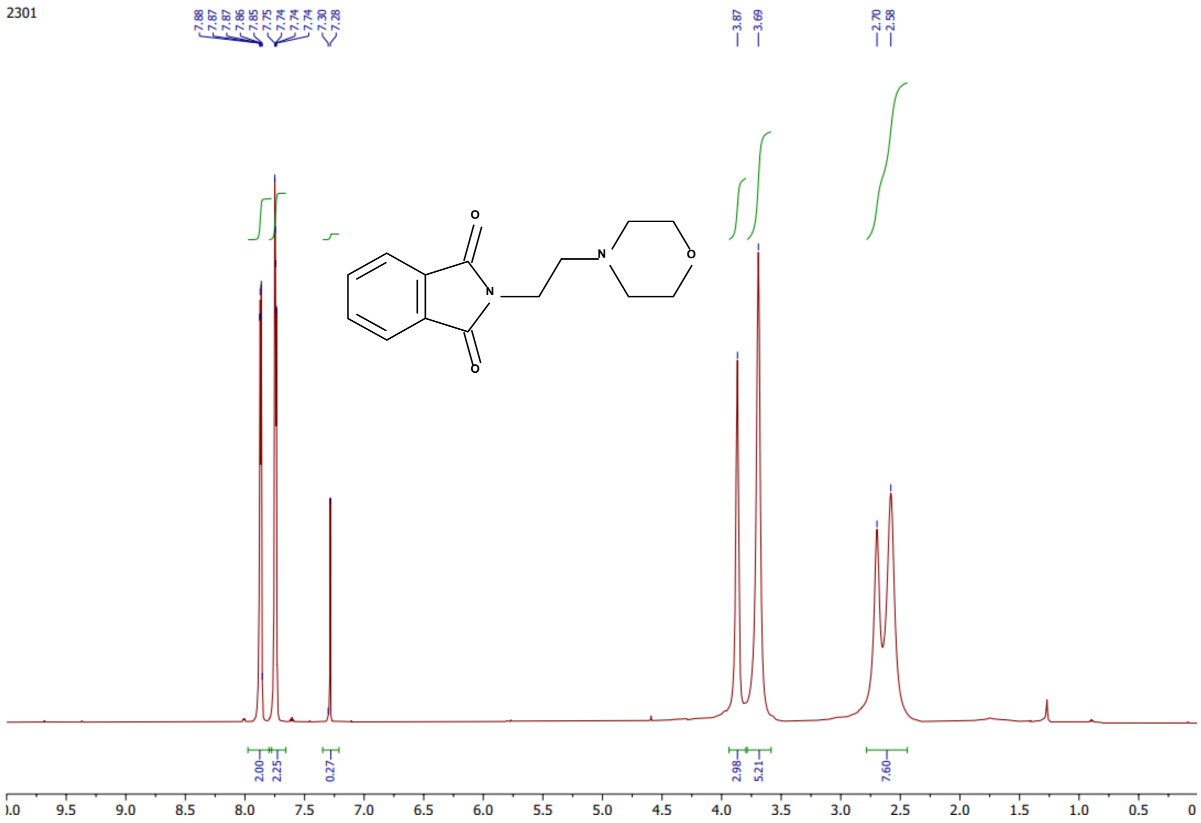


Figure S17: $^{1}$H NMR spectrum of the compound V

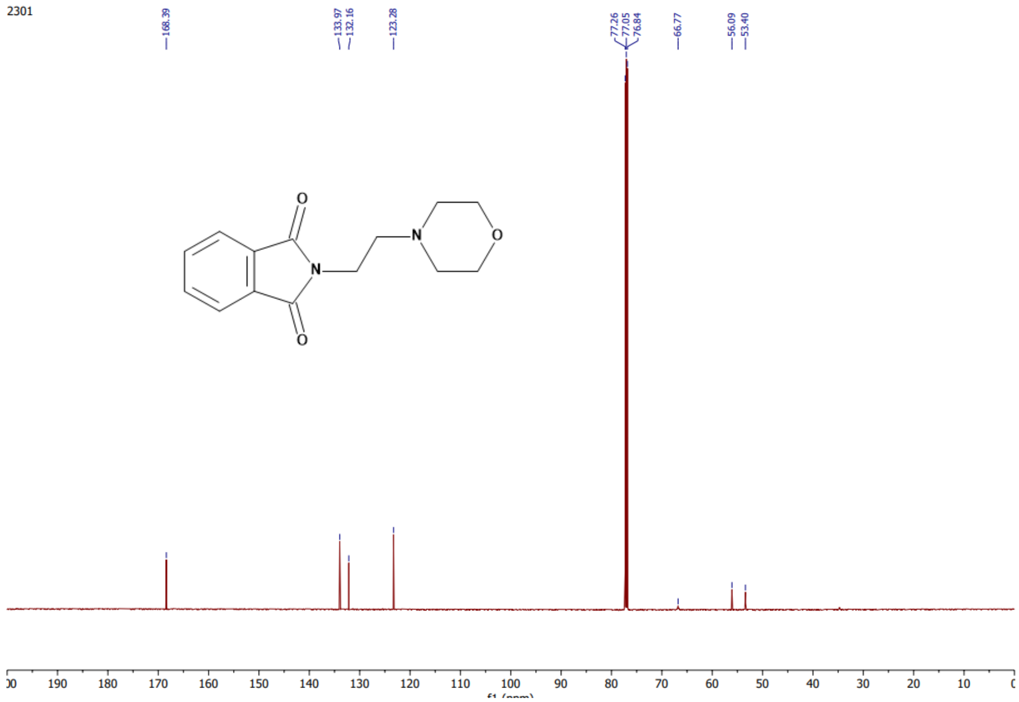


Figure S18: $^{13}$C NMR spectrum of the compound V

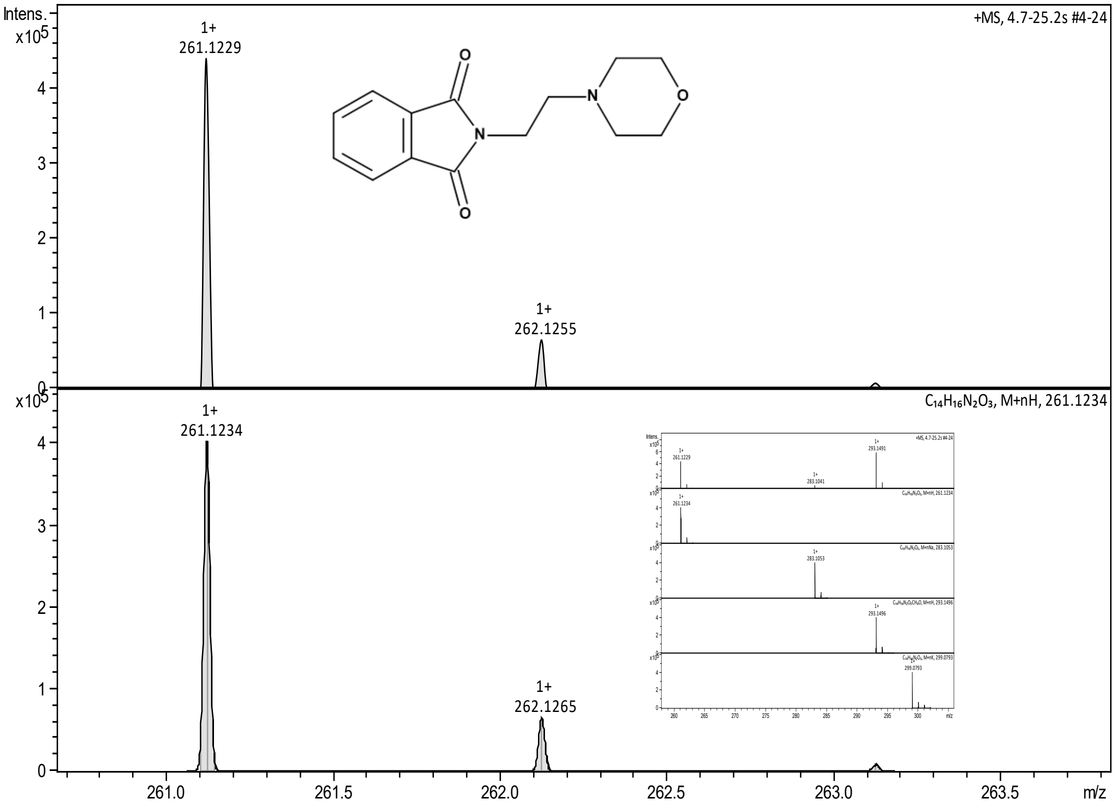


Figure S19: Mass spectrum of the compound V – method A.

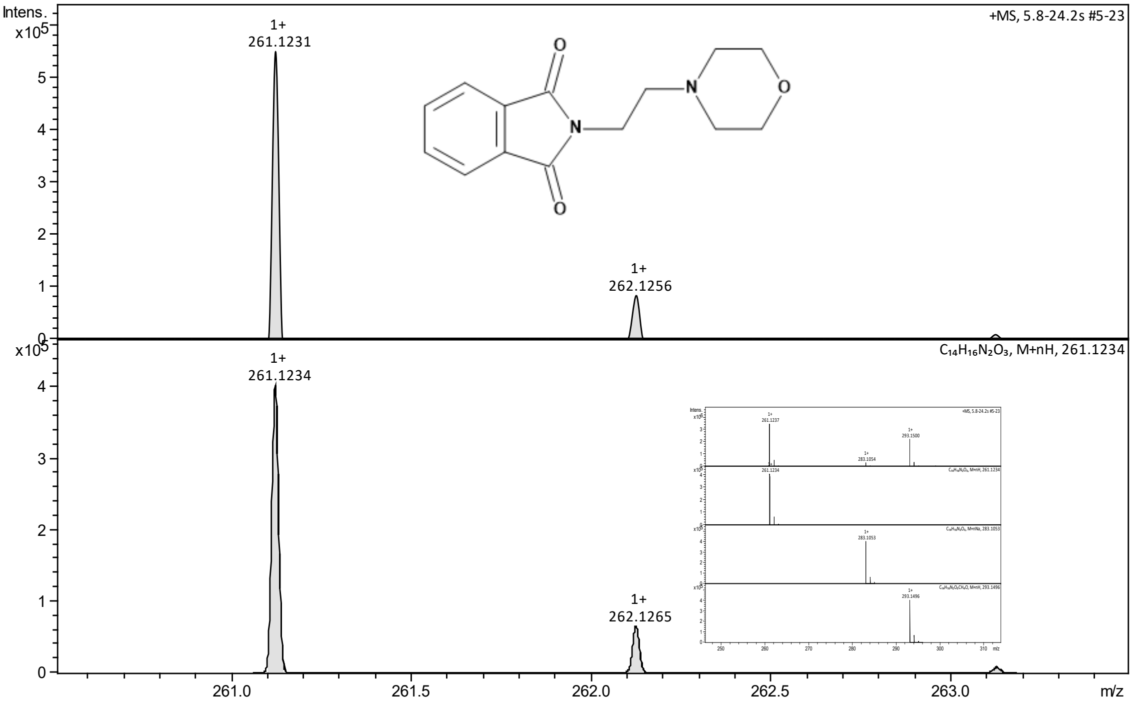

Figure S20: Mass spectrum of the compound V – method B.

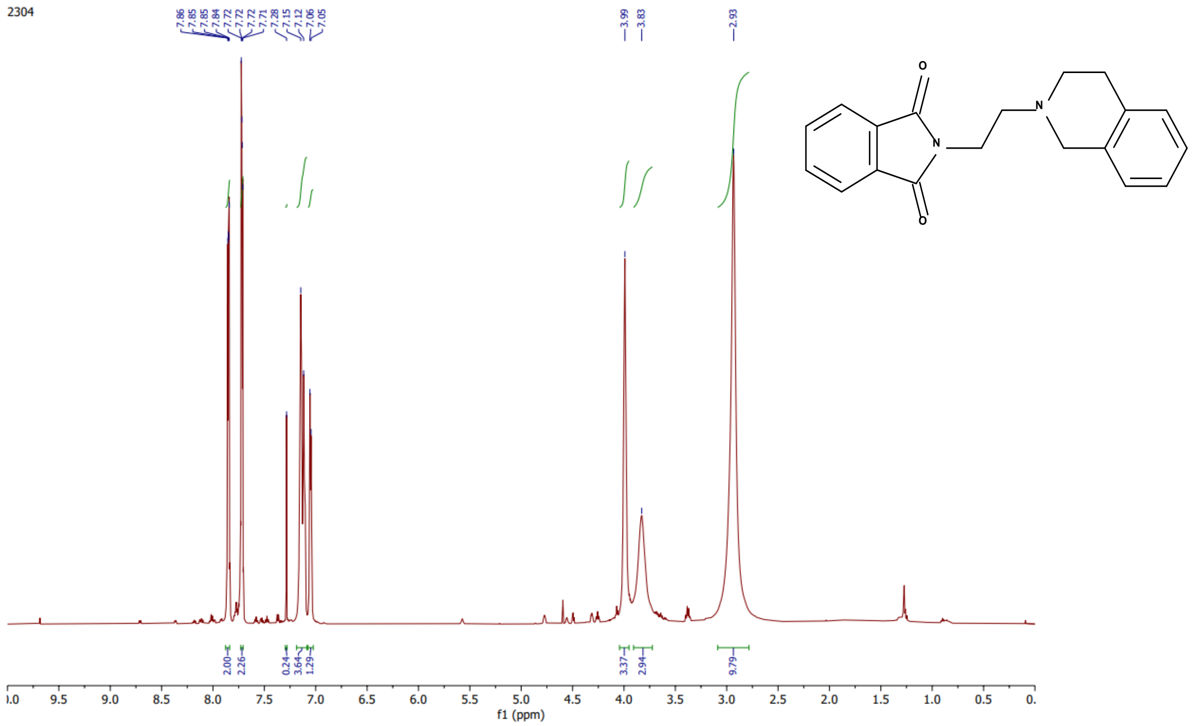


Figure S21: $^{1}$H NMR spectrum of the compound VI

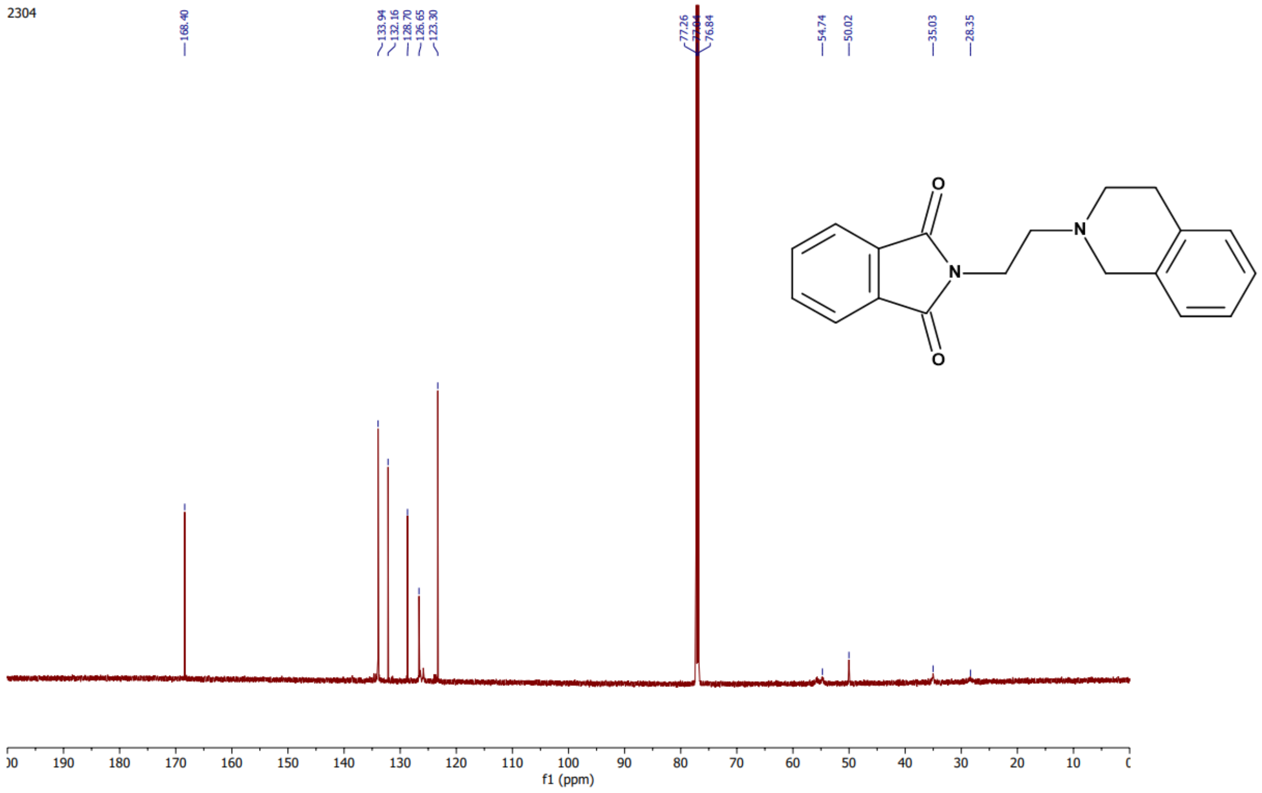

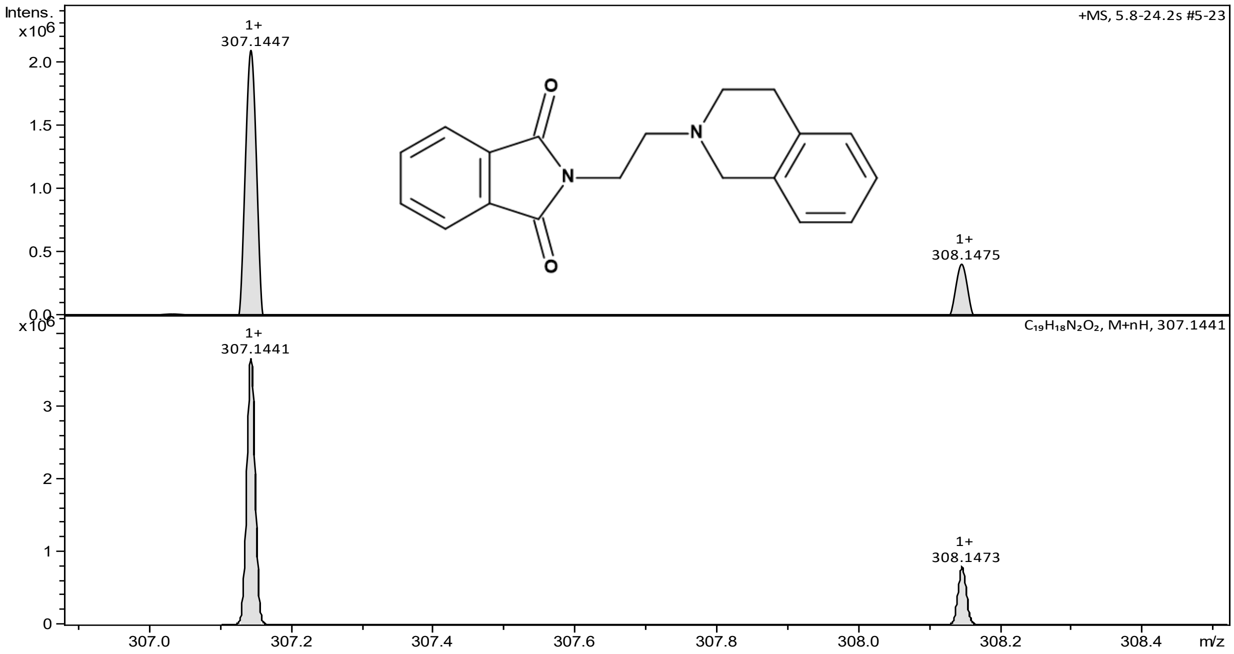


Figure S23: Mass spectrum of the compound VI.

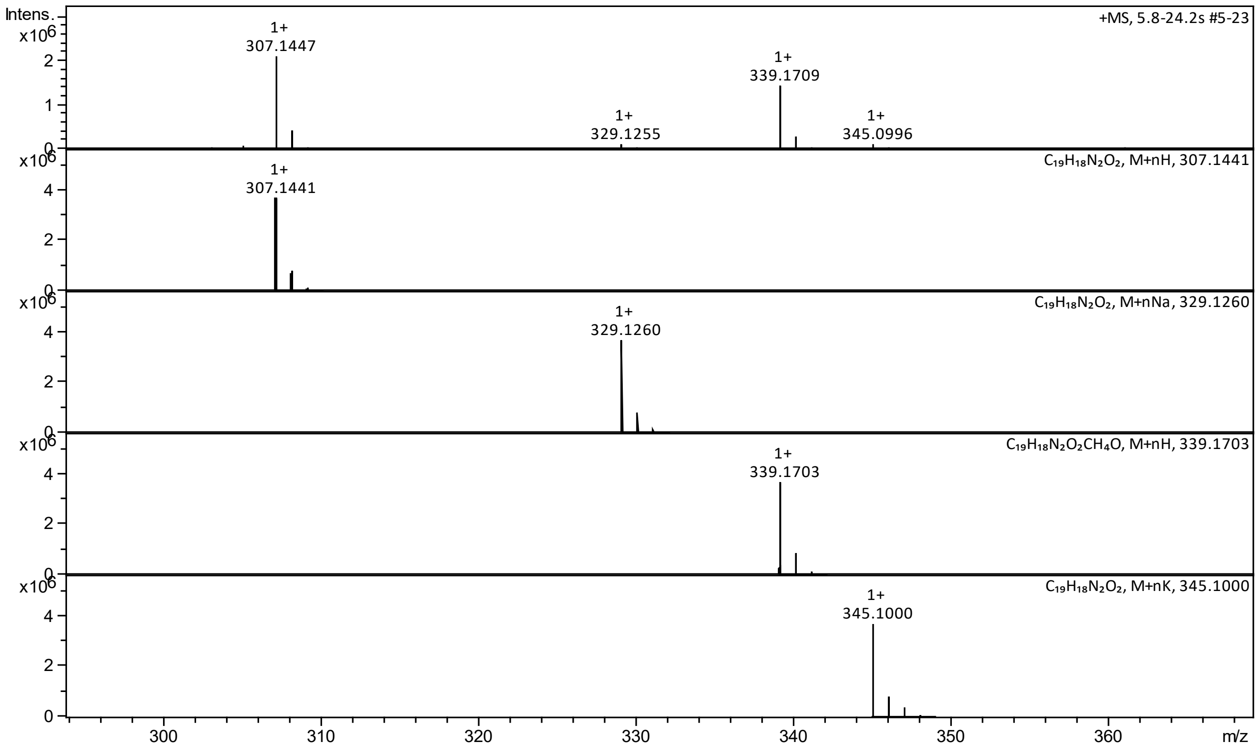


Figure S24: Mass spectrum of fragmentation of the compound VI.
